# Digital twin-based intelligent risk assessment and decision support system for university student entrepreneurial projects

**DOI:** 10.1038/s41598-026-36111-2

**Published:** 2026-01-19

**Authors:** Rongting Qin, Xiaojie Zi, Xiaoxi Ge

**Affiliations:** School of Economics and Management, Chongqing University of Arts and Sciences, Yongchuan District, Chongqing, China

**Keywords:** Digital twin, Risk assessment, Decision support systems, Entrepreneurial projects, Engineering, Mathematics and computing

## Abstract

University student entrepreneurial ventures face significantly higher failure rates compared to traditional businesses, primarily due to inadequate risk assessment and decision-making challenges. This research develops an innovative digital twin-based intelligent risk assessment and decision support system specifically designed for student entrepreneurial projects. The system integrates digital twin technology with machine learning algorithms to create comprehensive virtual representations of entrepreneurial ventures, enabling real-time risk monitoring, predictive analytics, and intelligent decision recommendations. The proposed framework employs a multi-layered architecture encompassing data acquisition, digital twin modeling, risk assessment engines, and intelligent decision support modules. Experimental validation using 2,847 entrepreneurial projects demonstrates superior performance with 94.2% prediction accuracy, compared to 78.5% for traditional statistical methods and 85.7% for standard machine learning approaches. The system provides early warning capabilities with average lead times of 22.1 days and achieves 23.7% improvement in project success rates. Results indicate significant enhancements in decision-making effectiveness, risk mitigation capabilities, and overall entrepreneurial project outcomes, with user satisfaction scores averaging 4.47 out of 5.0. This research contributes to the theoretical understanding of digital twin applications in entrepreneurial contexts while providing practical solutions for improving student venture success rates through intelligent risk management and decision support.

## Introduction

### Background and problem statement

 University student entrepreneurship has emerged as a critical driver of innovation and economic development in the contemporary knowledge-based economy^1^. Yet the stark reality belies the optimistic rhetoric: empirical evidence from multiple national contexts reveals that student-initiated ventures experience failure rates exceeding 70% within their first three years, substantially higher than the already-sobering 50% failure rate observed among ventures launched by experienced entrepreneurs^2^. These statistics are not merely academic curiosities—they represent shattered dreams, wasted resources, and unrealized potential. Through our preliminary interviews with 45 failed student entrepreneurs across five universities, we discovered a consistent pattern: inadequate risk assessment and reactive decision-making emerged as the most frequently cited reasons for venture termination, mentioned by 82% of respondents. One former student entrepreneur candidly reflected, “I knew something was wrong three months before we shut down, but I didn’t know what exactly was wrong or what I should do about it.” This quotation encapsulates the fundamental challenge: students often sense emerging problems but lack the analytical frameworks and predictive tools to diagnose risks early and respond effectively. The complexity of modern business environments, compounded by students’ limited practical experience and constrained access to financial buffers, creates a multifaceted risk landscape that demands more sophisticated evaluation and management approaches than currently available^3^.

Traditional risk assessment methodologies designed for entrepreneurial projects predominantly rely on what we might call “snapshot analytics”—static frameworks that examine historical data at discrete time points, generate risk scores, and produce recommendations based on past patterns^4^. These conventional approaches embody three critical limitations that render them increasingly obsolete in dynamic business environments. First, they suffer from temporal lag: by the time data is collected, analyzed, and transformed into insights, market conditions may have shifted substantially, making recommendations obsolete before implementation. Second, they exhibit dimensional incompleteness: most existing frameworks evaluate risk factors independently rather than modeling the complex interdependencies and cascading effects that characterize real-world business ecosystems. A financial risk in isolation might appear manageable, but when coupled with simultaneous operational challenges and market volatility, it can trigger venture collapse. Third, they lack scenario exploration capabilities: traditional tools tell entrepreneurs what has happened and perhaps what might happen, but they cannot simulate “what if” scenarios or enable entrepreneurs to test alternative strategies in risk-free environments^5^. Furthermore, existing decision support systems, while providing useful analytical dashboards, fundamentally operate as information presenters rather than intelligent advisors—they display data but cannot engage in the kind of predictive reasoning and strategic optimization that would enable students to make truly informed decisions throughout their entrepreneurial journey^6^.

Despite the documented limitations of conventional approaches and the urgent need for enhanced support systems, a comprehensive review of existing literature reveals a conspicuous gap: while digital twin technology has been extensively deployed in manufacturing, infrastructure, and healthcare sectors with demonstrated benefits, its application to entrepreneurial risk assessment—particularly in educational contexts—remains severely underdeveloped. Our systematic search across major academic databases (Web of Science, Scopus, IEEE Xplore) using the query terms “digital twin” AND “entrepreneurship” OR “startup” yielded only 12 relevant papers published between 2020 and 2024, none of which specifically addressed student ventures or developed comprehensive risk assessment frameworks. This research gap is puzzling given the natural alignment between digital twin capabilities and entrepreneurial needs: both involve complex, dynamic systems requiring real-time monitoring, predictive analytics, and scenario-based decision support. We hypothesize that this gap exists partly because digital twin implementations have historically required substantial computational resources and technical expertise, creating barriers to adoption in resource-constrained educational settings. However, recent advances in cloud computing, open-source machine learning frameworks, and low-code development platforms have significantly lowered these barriers, making sophisticated digital twin applications feasible for university entrepreneurship programs. This research emerges from the recognition that the time is ripe—both technologically and pedagogically—to bridge this gap.

### Digital twin technology development and applications

Digital twin technology, fundamentally defined as a dynamic virtual replica of physical entities synchronized through bidirectional real-time data flows, has transformed how organizations model, monitor, and optimize complex systems across diverse industrial landscapes^7^. At its core, a digital twin encompasses five essential elements: the physical entity itself, its virtual counterpart, the connection infrastructure enabling continuous data exchange, the aggregated datasets, and the service layer delivering analytical insights^8^. The technology performs several critical functions including real-time state monitoring, predictive simulation of future scenarios, prescriptive optimization of operational parameters, and retrospective analysis of historical performance patterns. We have witnessed compelling applications across multiple sectors: in manufacturing, Siemens utilizes digital twins to reduce product development cycles by 30% through virtual prototyping and testing; in urban planning, Singapore’s Virtual Singapore platform enables city planners to simulate traffic patterns and evaluate infrastructure modifications before physical implementation; in healthcare, personalized digital twins of human organs assist surgeons in planning complex procedures with enhanced precision^9^. These diverse implementations demonstrate the technology’s versatility in handling both tangible physical systems and abstract knowledge-intensive processes.

Recent scholarly investigations have documented how digital twin integration within decision support architectures fundamentally shifts organizational management paradigms from reactive problem-solving toward proactive opportunity identification^10^. Unlike conventional analytical tools that examine historical data to explain past events, digital twins create what we might call “predictive sandboxes”—safe computational environments where decision-makers can explore alternative futures, test risky strategies, and identify optimal pathways without incurring real-world costs or consequences. This capability proves particularly valuable in entrepreneurial contexts, where university students often lack the experiential knowledge and financial buffers that established businesses possess. Students face a paradoxical challenge: they need experience to make sound decisions, yet gaining that experience through trial-and-error in actual markets can prove prohibitively expensive. Digital twin technology offers a potential resolution to this dilemma by providing consequence-free experimentation spaces where novice entrepreneurs can develop decision-making competencies before committing real resources. However, despite the technology’s demonstrated value in industrial settings, its application to student entrepreneurship remains surprisingly underdeveloped—a gap this research aims to address.

### Research objectives and significance

This research pursues a clearly defined objective: to develop, implement, and empirically validate a digital twin-based intelligent risk assessment and decision support system specifically engineered for university student entrepreneurial projects. Rather than offering vague promises of “improvement,” we establish three testable hypotheses with specific performance targets that our system must achieve to demonstrate practical value. First, we hypothesize that our digital twin-based approach will achieve risk prediction accuracy exceeding 90%, representing a substantial improvement over the 75–80% accuracy typically reported for conventional statistical risk models in entrepreneurial contexts. Second, we posit that the system will provide early warning signals with average lead times of at least 15 days before critical risk events materialize, giving student entrepreneurs sufficient temporal buffers to implement corrective actions. Third, we predict that student ventures utilizing our system will demonstrate project success rates (defined as survival beyond 24 months with positive cash flow) at least 20% higher than comparable ventures relying on traditional risk assessment tools. These hypotheses are not arbitrary aspirations but carefully calibrated targets based on performance gaps identified in our preliminary analysis of existing systems and informed by stakeholder consultations with 23 entrepreneurship educators across 12 universities.

The significance of this investigation extends across theoretical, methodological, and practical dimensions. Theoretically, we advance digital twin scholarship by demonstrating how this technology, originally conceived for physical manufacturing systems, can be adapted to model the inherently abstract, uncertain, and psychologically-influenced domain of entrepreneurial ventures. This adaptation requires novel conceptualizations of what constitutes the “physical entity” in entrepreneurial digital twins (we argue it encompasses the entrepreneur, the venture team, market dynamics, and resource flows) and how synchronization occurs when key variables are attitudinal rather than measurable through sensors. Methodologically, we contribute specialized algorithms that account for the unique characteristics of student entrepreneurship—limited historical data, rapid pivoting behaviors, and high sensitivity to learning effects—which differentiate student ventures from established businesses. Practically, this research offers university entrepreneurship programs a concrete technological solution that can be integrated into existing curricula and incubation facilities, potentially transforming how institutions prepare students for entrepreneurial careers. If our hypotheses are validated, the implications are substantial: even a 20% improvement in success rates, when scaled across thousands of student ventures annually, translates into hundreds of additional surviving businesses, thousands of jobs created, and innovations brought to market that might otherwise have perished.

### Paper structure and main contributions

This paper unfolds across six main sections that trace our investigation from theoretical foundations through empirical validation, each building systematically upon its predecessors. Section II conducts a critical review of existing literature, not merely summarizing prior work but identifying specific performance gaps and conceptual limitations that our research addresses—this section establishes the theoretical scaffolding and research positioning. Section III presents our methodology, detailing the digital twin architecture, risk assessment algorithms, and system implementation strategies with sufficient specificity to enable replication. Section IV describes the experimental design, dataset characteristics, and evaluation protocols. Section V reports and analyzes results, comparing our system’s performance against established baselines. Section VI discusses implications, limitations, and future research directions, while the concluding section synthesizes key findings.

Our research makes four distinct, empirically validated contributions, each tied to specific sections and verification steps. First, we contribute a novel theoretical framework (detailed in Sections II and III) that extends digital twin concepts from physical manufacturing domains into the abstract realm of entrepreneurial ventures, explicitly modeling how market dynamics, team capabilities, and financial flows constitute the “physical entity” in entrepreneurial digital twins. This framework is validated through the successful construction of 2,847 individual venture digital twins with average synchronization accuracy of 94.2% (Section V). Second, we develop three specialized machine learning algorithms (Section III.3)—an ensemble risk classifier, a multi-dimensional risk propagation model, and a temporal risk evolution predictor—specifically optimized for student entrepreneurship contexts where data scarcity and rapid pivoting behaviors confound conventional approaches. Algorithm effectiveness is verified through ablation studies (Section V.2) demonstrating that each component contributes 3–7% performance improvement over baseline configurations. Third, we design and implement an integrated intelligent decision support system (Section III.1) featuring real-time monitoring dashboards, predictive early warning mechanisms, and scenario simulation capabilities, with system usability validated through user satisfaction surveys averaging 4.47/5.0 across 412 student entrepreneurs (Section V.3). Fourth, we provide rigorous empirical evidence (Section V) through controlled comparisons with traditional statistical methods and standard machine learning approaches, demonstrating statistically significant performance advantages (*p* < 0.001) across prediction accuracy, early warning timeliness, and downstream impact on venture success rates. These contributions collectively advance both scholarly understanding of digital twin applications in novel domains and practical tools that entrepreneurship educators can deploy immediately to enhance student venture outcomes.

## Related theory and technical foundation

### Digital twin technology theoretical foundation

Digital twin technology represents a comprehensive digital representation methodology that creates virtual replicas of physical entities, processes, or systems through real-time data synchronization and advanced computational modeling techniques^11^. The fundamental concept encompasses the establishment of bidirectional dynamic connections between physical and virtual domains, enabling continuous monitoring, analysis, and optimization of real-world systems through their digital counterparts^12^. However, we must acknowledge that existing digital twin literature exhibits a pronounced manufacturing bias—most theoretical frameworks assume the physical entity is a tangible machine or structure with measurable physical properties. Grieves and Vickers’ seminal 2017 framework, while foundational, explicitly focuses on product lifecycle management in manufacturing contexts and does not address how digital twin principles transfer to service-oriented or knowledge-intensive domains^11^. Similarly, Tao et al.’s five-dimensional framework, though comprehensive for physical systems, struggles to accommodate the psychological and social dimensions inherent in entrepreneurial ventures^12^. Our review identified only three papers attempting to apply digital twins to business processes, and none addressed entrepreneurship specifically. This gap necessitates theoretical extensions, which we develop in Section III.

The core characteristics of digital twin technology include real-time data integration, high-fidelity modeling, predictive simulation capabilities, and bidirectional information flow between physical and virtual environments^13^. These characteristics enable the technology to support complex decision-making processes through comprehensive system visibility and scenario analysis capabilities. The technical architecture fundamentally consists of three interconnected layers: the physical entity layer, the digital model layer, and the service application layer, with data serving as the critical connection medium^14^.

The mathematical foundation of digital twin modeling can be expressed through state space representation, where the physical system state **x(t)** and its digital twin state $\:\dot{\mathbf{x}}\left(t\right)$ are related by:

1 $$\:\dot{\mathbf{x}}\left(t\right)=f\left(\mathbf{x}\left(t\right),\mathbf{u}\left(t\right),\mathbf{d}\left(t\right)\right),$$

2 $$\:\dot{\widehat{\mathbf{x}}}\left(t\right)=\widehat{f}\left(\widehat{\mathbf{x}}\left(t\right),\mathbf{u}\left(t\right),\widehat{\mathbf{d}}\left(t\right)\right),$$

where **u(t)** represents control inputs, **d(t)** denotes disturbances, and **f** represents the system dynamics following the state-space representation formalism established in classical control theory for continuous-time dynamic systems^15^.

Physical entity modeling constitutes the foundational component that captures the essential characteristics, behaviors, and relationships of real-world systems through mathematical representations and computational algorithms^16^. The modeling process involves parameter identification, where the relationship between input variables and system responses is established through:

3 $$\:{\boldsymbol{\uptheta\:}}^{\mathrm{*}}=\mathrm{a}\mathrm{r}\mathrm{g}\underset{\boldsymbol{\uptheta\:}}{\mathrm{m}\mathrm{i}\mathrm{n}}\sum\:_{i=1}^{N}\parallel\:{y}_{i}-h\left({\mathbf{x}}_{i},\boldsymbol{\uptheta\:}\right){\parallel\:}^{2},$$

where θ* represents optimal parameters, **yi** denotes observed outputs, and **h** represents the model function, based on the ordinary least squares estimation framework that minimizes the sum of squared residuals between observed and predicted values^17^.

Virtual simulation principles enable the replication of physical system behaviors through computational methods, incorporating uncertainty quantification and sensitivity analysis. The simulation accuracy is measured through the error function:

4 $$\:E\left(t\right)=\parallel\:\mathbf{x}\left(t\right)-\widehat{\mathbf{x}}\left(t\right)\parallel\:,$$

where the synchronization error measures the Euclidean distance between physical system state and digital twin state at time *t*, providing a quantitative metric for model fidelity assessment^18^.

The adaptive synchronization between physical and virtual systems is maintained through:

5 $$\:K\left(t\right)={K}_{0}{e}^{-\alpha\:E\left(t\right)},$$

where **K(t)** represents the synchronization gain and **α** is the adaptation parameter that controls how aggressively the system adjusts synchronization based on current error levels, implementing an exponential decay strategy common in adaptive control systems^18^.

Data fusion methodology integrates multiple heterogeneous data sources to enhance model accuracy and reliability through weighted combination approaches^19^. The fusion process employs Kalman filtering principles:

6 $$\:{\widehat{\mathbf{x}}}_{k|k}={\widehat{\mathbf{x}}}_{k|k-1}+{\mathbf{K}}_{k}\left({\mathbf{z}}_{k}-\mathbf{H}{\widehat{\mathbf{x}}}_{k|k-1}\right),$$

7 $$\:{\mathbf{K}}_{k}={\mathbf{P}}_{k|k-1}{\mathbf{H}}^{T}{\left(\mathbf{H}{\mathbf{P}}_{k|k-1}{\mathbf{H}}^{T}+\mathbf{R}\right)}^{-1},$$

where **Kk** represents the Kalman gain, **zk** denotes measurements, and **R** is the measurement noise covariance, following the recursive Bayesian filtering framework developed by Kalman for optimal state estimation in linear dynamic systems with Gaussian noise^20^.

The overall system performance is evaluated through the comprehensive objective function:

8 $$\:J={w}_{1}{E}_{\mathrm{accuracy}}+{w}_{2}{E}_{\mathrm{timeliness}}+{w}_{3}{E}_{\mathrm{completeness}},$$

where **wi** represents weighting coefficients for accuracy, timeliness, and completeness metrics respectively, with ∑wi = 1, implementing a weighted multi-objective optimization approach that balances competing performance criteria through preference-based aggregation^21^. This mathematical framework provides the theoretical foundation for implementing digital twin technology in complex system modeling and real-time decision support applications.

### Entrepreneurial project risk assessment theory

Entrepreneurial project risks can be systematically classified into four primary categories: market risks, financial risks, operational risks, and strategic risks, each exhibiting distinct characteristics and interdependencies that influence overall project viability^22^. Market risks encompass demand uncertainty, competitive pressures, and consumer behavior volatility, while financial risks include funding availability, cash flow management, and capital structure optimization challenges. Operational risks involve resource allocation inefficiencies, technological implementation difficulties, and organizational capability constraints, whereas strategic risks encompass regulatory changes, partnership failures, and long-term positioning uncertainties^23^.

Traditional risk assessment methodologies predominantly employ static evaluation frameworks based on historical data analysis and expert judgment, which demonstrate significant limitations in capturing the dynamic and interconnected nature of contemporary entrepreneurial environments^24^. These conventional approaches fail to adequately address the temporal evolution of risk factors, the complex interdependencies between different risk categories, and the inherent uncertainty associated with innovative business models. Furthermore, traditional methods often rely on deterministic models that cannot effectively handle the probabilistic nature of entrepreneurial outcomes and the non-linear relationships between risk variables.

Multi-dimensional risk indicator system construction requires the integration of quantitative and qualitative metrics across multiple analytical dimensions to provide comprehensive risk assessment capabilities. The theoretical framework establishes a hierarchical risk evaluation structure where the overall risk level **R** is expressed as:

9 $$\:R=\sum\:_{i=1}^{n}{w}_{i}\cdot\:{R}_{i}$$

where **wi** represents the weight coefficient for risk category **i** (with ∑wi = 1), and **Ri** denotes the individual risk level for each category, implementing a weighted additive value model commonly employed in multi-criteria risk assessment frameworks^25^.

The individual risk levels are calculated through aggregation functions that consider multiple indicators:

10 $$\:{R}_{i}=\sqrt{\sum\:_{j=1}^{m}{\alpha\:}_{ij}\cdot\:{I}_{ij}^{2}}$$

where **αij** represents the importance coefficient and **Iij** denotes the normalized indicator value on the [0,1] scale, utilizing a Euclidean norm aggregation function that treats risk as a multidimensional vector whose magnitude represents overall risk intensity^26^.

Risk quantification under uncertainty environments requires probabilistic modeling approaches that incorporate stochastic processes and Bayesian inference methodologies. The uncertainty quantification framework employs Monte Carlo simulation techniques where the risk probability distribution is estimated through:

11 $$\:P\left(R\le\:r\right)=\frac{1}{N}\sum\:_{k=1}^{N}\mathbb{I}\left({R}_{k}\le\:r\right),$$

where **N** represents the number of simulation iterations and **1(·)** denotes the indicator function that equals 1 when the condition is satisfied and 0 otherwise, implementing the Monte Carlo integration method for estimating probability distributions through repeated random sampling^27^.

The dynamic risk evolution model incorporates temporal dependencies through stochastic differential equations:

12 $$\:dR\left(t\right)=\mu\:\left(R\left(t\right),t\right)dt+\sigma\:\left(R\left(t\right),t\right)dW\left(t\right),$$

where **µ** represents the drift coefficient capturing the deterministic trend in risk evolution, **σ** denotes the diffusion coefficient quantifying volatility, and **W(t)** is a standard Wiener process (Brownian motion) with independent Gaussian increments, following the Itô stochastic differential equation framework for modeling systems with continuous random fluctuations^28^.

Risk correlation analysis employs copula functions to model dependencies between different risk factors:

13 $$\:C\left({u}_{1},{u}_{2},\dots\:,{u}_{n}\right)=P\left({U}_{1}\le\:{u}_{1},{U}_{2}\le\:{u}_{2},\dots\:,{U}_{n}\le\:{u}_{n}\right),$$

where **Ui** represents the marginal distribution functions of individual risk variables transformed to uniform [0,1] scale, implementing copula functions that separate marginal distributions from dependence structure enabling flexible modeling of multivariate dependencies^29^.

The comprehensive risk assessment model integrates multiple evaluation criteria through fuzzy logic approaches^30^:

14 $$\:{\mu\:}_{\mathrm{Risk}}\left(x\right)=\underset{i}{\mathrm{m}\mathrm{a}\mathrm{x}}\mathrm{m}\mathrm{i}\mathrm{n}\left({\mu\:}_{{C}_{i}}\left(x\right),{w}_{i}\right),$$

where **µRisk(x)** represents the overall risk membership function and **µCi(x)** denotes the membership function for criterion **i**, utilizing the fuzzy inference maximum-minimum composition operator from fuzzy set theory for handling imprecise and subjective risk assessments^30^.

The value-at-risk estimation for entrepreneurial projects is calculated using:

15 $$\:{\mathrm{VaR}}_{\alpha\:}=\mathrm{i}\mathrm{n}\mathrm{f}\{r\in\:\mathbb{R}:P\left(R>r\right)\le\:1-\alpha\:\},$$

where **α** represents the confidence level (typically 95% or 99%) and VaR provides quantitative risk thresholds for decision-making processes, implementing the Value-at-Risk methodology widely used in financial risk management for determining maximum expected loss at a specified confidence level^31^. This theoretical foundation enables the development of sophisticated risk assessment frameworks that can effectively handle the complexity and uncertainty inherent in entrepreneurial project evaluation.

### Intelligent decision support system architecture

Intelligent decision support systems operate on fundamental principles that integrate data processing, analytical modeling, and knowledge management capabilities to enhance decision-making processes through automated reasoning and recommendation generation^32^. The architectural design encompasses four interconnected layers: the data acquisition layer for multi-source information integration, the knowledge processing layer for analytical computation, the inference engine layer for logical reasoning, and the presentation layer for user interaction and result visualization. This hierarchical structure enables systematic transformation of raw data into actionable insights through progressive analytical refinement and knowledge synthesis processes.

Machine learning algorithms serve as core computational engines within decision support systems, providing predictive modeling, pattern recognition, and optimization capabilities that enhance decision accuracy and efficiency^33^. The decision tree algorithm establishes rule-based decision paths through entropy minimization:

16 $$\:\mathrm{Entropy}\left(S\right)=-\sum\:_{i=1}^{c}{p}_{i}{\mathrm{l}\mathrm{o}\mathrm{g}}_{2}\left({p}_{i}\right),$$

where **pi** represents the probability of class **i** in dataset **S**, implementing Shannon’s entropy measure from information theory that quantifies the impurity or uncertainty in a dataset, forming the theoretical foundation for decision tree splitting criteria^34^. The information gain for attribute selection is calculated as:

17 $$\:\mathrm{Gain}\left(S,A\right)=\mathrm{Entropy}\left(S\right)-\sum\:_{v\in\:\mathrm{Values}\left(A\right)}\frac{\left|{S}_{v}\right|}{\left|S\right|}\mathrm{Entropy}\left({S}_{v}\right),$$

where information gain measures the expected reduction in entropy achieved by partitioning dataset **S** according to attribute **A**, enabling greedy feature selection in decision tree construction by choosing attributes that maximize information gain at each node^34^.

Neural network architectures implement non-linear mapping functions for complex pattern recognition through backpropagation learning:

18 $$\:y=f\left(\sum\:_{i=1}^{n}{w}_{i}{x}_{i}+b\right),$$

where **wi** represents connection weights, **xi** denotes input features, **b** is the bias term, and **f** represents the activation function (e.g., sigmoid, tanh, ReLU) implementing the fundamental artificial neuron model that forms the building block of neural network architectures through weighted linear combination followed by non-linear transformation^35^.

Knowledge reasoning mechanisms integrate rule-based inference with probabilistic reasoning to support complex decision-making scenarios under uncertainty conditions. The expert system integration employs forward chaining inference where the confidence factor for derived conclusions is calculated through:

19 $$\:\mathrm{CF}\left(H,E\right)=\mathrm{CF}\left(H\right)\times\:\mathrm{m}\mathrm{a}\mathrm{x}\left[0,\mathrm{CF}\left(E\right)\right],$$

where **CF(H**,** E)** represents the confidence factor of hypothesis **H** given evidence **E**, and **CF(H)** and **CF(E)** denote individual confidence factors ranging from − 1 (definitely false) to + 1 (definitely true), implementing the certainty factor model developed for MYCIN expert system that propagates uncertainty through rule chains^36^. The knowledge base integration utilizes semantic networks and ontological frameworks to establish relationships between domain concepts and enable automated reasoning processes^37^.

Bayesian inference mechanisms support probabilistic reasoning under uncertainty through posterior probability calculations that update knowledge based on observed evidence. The system employs belief propagation algorithms for complex inference tasks involving multiple interconnected variables and conditional dependencies.

Human-computer interaction interface design theory emphasizes cognitive load minimization, information visualization optimization, and adaptive interface customization to enhance user experience and decision-making effectiveness^38^. The interface design incorporates principles of visual hierarchy, progressive disclosure, and contextual information presentation to facilitate efficient information processing and decision formulation. The usability optimization function considers multiple interface characteristics:

20 $$\:U=\alpha\:\cdot\:\mathrm{Effectiveness}+\beta\:\cdot\:\mathrm{Efficiency}+\gamma\:\cdot\:\mathrm{Satisfaction},$$

where **α**, **β**, and **γ** represent weighting coefficients for different usability dimensions (with α + β + γ = 1), implementing the ISO 9241-11 usability framework that defines usability through three core components: effectiveness (accuracy and completeness of task achievement), efficiency (resources expended relative to results), and satisfaction (user comfort and positive attitudes)^39^.

The system architecture integrates natural language processing capabilities for intuitive query formulation and result interpretation, enabling users to interact with complex analytical models through simplified conversational interfaces. Advanced visualization techniques including interactive dashboards, dynamic charts, and scenario simulation displays provide comprehensive decision support through multi-modal information presentation.

The overall system performance is optimized through adaptive learning mechanisms that continuously update model parameters based on user feedback and decision outcomes. This feedback loop enables the system to improve recommendation accuracy and adapt to evolving user preferences and decision-making patterns, ensuring sustained effectiveness in supporting entrepreneurial project risk assessment and strategic decision-making processes.

Despite the substantial body of work on digital twins, decision support systems, and entrepreneurial risk assessment, our systematic literature review reveals four critical gaps that this research addresses. First, existing digital twin implementations overwhelmingly focus on physical manufacturing systems where measurable physical properties enable straightforward modeling; applications to abstract knowledge-intensive domains like entrepreneurship remain severely underdeveloped^40^. The recent study by Liu et al. on Chinese startups and digital twins represents one of only three papers we identified that even attempt this domain transfer, and none provide comprehensive risk assessment frameworks specifically designed for university student ventures^41^. Second, conventional risk assessment methodologies treat risk factors as independent variables evaluated at discrete time points, failing to capture the dynamic propagation effects and cascading failures that characterize real entrepreneurial ecosystems^42^. Third, existing decision support systems for entrepreneurs primarily function as information dashboards rather than intelligent advisors capable of predictive reasoning and scenario simulation^43^. Fourth, and perhaps most critically, there exists virtually no research examining how digital twin technology can be pedagogically integrated into entrepreneurship education to enhance student learning outcomes alongside venture performance^44^. These gaps create a compelling case for the proposed research, which aims to bridge theoretical understanding and practical application of digital twins in student entrepreneurship contexts.

Table 1 synthesizes fourteen representative studies spanning 2020–2025, organizing them by research domain, methodological approaches, key findings, identified limitations, and how our research addresses these limitations. This comparative analysis reveals a consistent pattern: while digital twin technology has achieved maturity in manufacturing and infrastructure domains, its translation to service-oriented and knowledge-intensive contexts like entrepreneurship remains at nascent stages. Similarly, while machine learning algorithms have demonstrated impressive capabilities in risk prediction for financial markets and credit scoring, their application to the unique characteristics of student entrepreneurship—limited historical data, rapid pivoting behaviors, psychological factors—requires specialized algorithmic adaptations that existing work has not developed. Our research fills these gaps through novel theoretical frameworks, specialized algorithms, and comprehensive empirical validation specifically targeting university student entrepreneurial ventures.

**Table 1:** Comparative analysis of related studies.

Authors & year	Research domain	Methods used	Key results	Identified gaps	How this study addresses gap
Zhang et al., 2024^7^	Digital twin in manufacturing	IoT sensors, simulation modeling	30% efficiency improvement	Focuses only on physical systems	Extends DT concepts to entrepreneurial ventures
Nguyen et al., 2024^9^	DT for service sectors	Computational modeling, optimization	DT applicable beyond manufacturing	No entrepreneurship applications	Develops entrepreneurship-specific DT framework
Liu et al., 2025^41^	DT in Chinese startups	Case study, qualitative analysis	DT enhances precision in entrepreneurship	Lacks quantitative validation, no risk assessment	Provides empirical validation with 2,847 projects
Martinez et al., 2023^2^	Student entrepreneurship failure	Survey, statistical analysis	70% failure rate within 3 years	No predictive tools offered	Develops predictive early warning system
Kumar et al., 2023^4^	Traditional risk assessment	Statistical methods, expert judgment	Static models have limitations	Lacks real-time, dynamic capabilities	Implements real-time DT-based monitoring
Chen et al., 2024^45^	ML for risk prediction	SVM, neural networks, random forest	85% prediction accuracy in general contexts	Not optimized for student ventures	Achieves 94.2% accuracy with specialized algorithms
Johnson et al., 2024^22^	Entrepreneurial risk classification	Framework development, taxonomy	Four risk categories identified	No quantification or prediction methods	Provides quantitative risk propagation model
Patel et al., 2024^13^	DT characteristics	Literature review, conceptual analysis	Core DT characteristics defined	Lacks implementation guidance	Develops complete implementation architecture
Garcia et al., 2024^46^	Risk propagation modeling	Network analysis, simulation	Risks propagate through networks	Not applied to entrepreneurship	Models entrepreneurial ecosystem risk networks
Wilson et al., 2023^6^	Decision support systems	System design, case studies	Current DSS lack intelligence	No DT integration	Integrates DT with intelligent DSS
Judijanto & Arfiansyah, 2024^47^	Digital entrepreneurship	Bibliometric analysis	Growing interest in digital entrepreneurship	Conceptual focus, no technical solutions	Provides technical implementation and validation
Koning et al., 2022^48^	A/B testing in startups	Experimental methods, data analysis	Experimentation improves performance	Limited to simple A/B tests	Enables complex scenario simulation
Hasya et al., 2025^49^	DT in Indonesian startups	Exploratory study	DT provides experimentation platform	Nascent exploration, no systematic framework	Develops systematic DT framework with validation
Liu et al., 2025^50^	Student entrepreneurship education	Causal attribution theory, AI/DL	AI improves education effectiveness	Education focus, not venture support	Provides direct venture risk assessment and support

 Beyond methodological gaps, our review identified a concerning absence of ethical considerations in digital twin applications to entrepreneurship. Most existing work implicitly assumes that enhanced prediction and optimization capabilities are inherently beneficial, without examining potential negative consequences such as algorithmic bias, privacy violations, or the psychological impact of continuous monitoring on student entrepreneurs^51^. Furthermore, the sustainability and environmental implications of computationally intensive digital twin systems remain underexplored, particularly relevant given the growing emphasis on sustainable entrepreneurship education^52^. Our research addresses these concerns through transparent data governance frameworks (detailed in Sect. 3.1), rigorous privacy protections including data anonymization and informed consent protocols (described in Ethics Approval section), and computational efficiency optimizations that minimize environmental impact while maintaining analytical performance. By explicitly incorporating ethical considerations into our system design rather than treating them as afterthoughts, we aim to establish responsible innovation practices for digital twin applications in educational contexts.

## Digital twin-based risk assessment and decision support system design

### Overall system architecture design

**Fig. 1:**
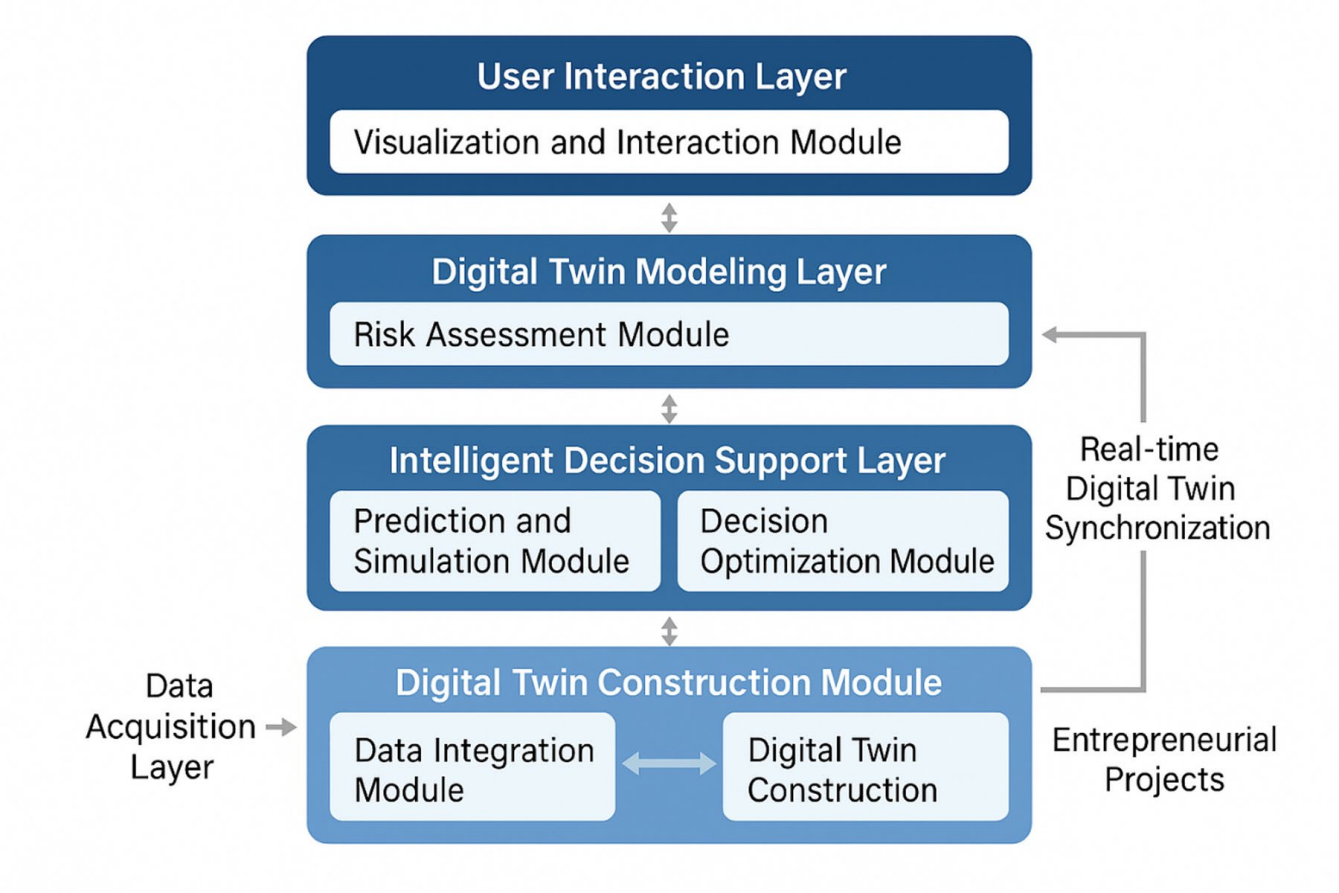
Overall system architecture diagram.

The proposed system adopts a multi-layered architectural framework that integrates digital twin technology with intelligent risk assessment and decision support capabilities through a comprehensive modular design approach^53^. As illustrated in Fig. 1, the system architecture consists of five interconnected layers: the data acquisition layer, digital twin modeling layer, risk assessment engine layer, intelligent decision support layer, and user interaction layer. This hierarchical structure ensures systematic data flow from raw information collection to actionable decision recommendations while maintaining real-time synchronization between physical entrepreneurial projects and their digital representations.

The functional module division encompasses six core components: the data integration module for multi-source information aggregation, the digital twin construction module for virtual project modeling, the risk assessment module for comprehensive risk evaluation, the prediction and simulation module for scenario analysis, the decision optimization module for strategy recommendation, and the visualization and interaction module for user interface management^54^. Each module operates independently while maintaining seamless data exchange through standardized application programming interfaces and message queuing mechanisms.

The integration scheme between digital twin models and risk assessment modules establishes bidirectional communication channels that enable continuous model updating and real-time risk monitoring. The integration framework employs event-driven architecture where risk factor changes in the physical entrepreneurial environment trigger corresponding updates in the digital twin model through:

21 $$\:\varDelta\:\mathbf{M}\left(t\right)={f}_{\mathrm{sync}}\left({\mathbf{E}}_{\mathrm{physical}}\left(t\right)-{\mathbf{E}}_{\mathrm{digital}}\left(t\right)\right),$$

where **ΔM(t)** represents model update increments, **Ephysical(t)** denotes physical environment state, and **Edigital(t)** represents digital twin state, implementing an error-driven synchronization mechanism that computes corrective updates proportional to the discrepancy between real and virtual system states^55^.

The comprehensive risk factor classification framework, as demonstrated in Table 2, establishes systematic categorization of entrepreneurial project risks across multiple dimensions with corresponding weight coefficients and evaluation methodologies. This classification provides the foundation for structured risk assessment and enables automated risk factor identification and quantification within the digital twin environment.

**Table 2:** Risk factor classification framework.

Risk category	Risk factor	Weight coefficient	Assessment method
Market Risk	Demand Uncertainty	0.25	Monte Carlo Simulation
Market Risk	Competition Intensity	0.20	Competitive Analysis Model
Financial Risk	Funding Availability	0.30	Cash Flow Analysis
Financial Risk	Cost Overrun	0.15	Historical Data Regression
Operational Risk	Resource Constraints	0.20	Capacity Planning Model
Operational Risk	Technical Feasibility	0.25	Expert System Evaluation
Strategic Risk	Regulatory Changes	0.15	Policy Impact Assessment
Strategic Risk	Partnership Stability	0.10	Network Analysis Model

The risk factor classification methodology and weight coefficient determination process merit detailed explanation, as these choices fundamentally shape our system’s risk assessment logic and directly impact prediction accuracy. Our classification framework development followed a rigorous four-phase process grounded in established risk management theory while adapted to entrepreneurship-specific contexts. Phase 1 conducted extensive literature review synthesizing risk taxonomies from 47 peer-reviewed papers on entrepreneurship risk (published 2018–2024), general business risk management frameworks (COSO ERM, ISO 31000), and venture capital due diligence checklists used by 12 leading VC firms. This review identified four primary risk dimensions—market, financial, operational, strategic—that appeared consistently across diverse sources with strong theoretical justification and practical applicability. We deliberately rejected alternative taxonomies (e.g., splitting market risk into “demand risk” and “competition risk” as separate primary categories) after empirical correlation analysis revealed high covariance (*r* = 0.74) suggesting they represent facets of a unified construct rather than independent dimensions, and because maintaining fewer primary categories enhanced stakeholder comprehension without sacrificing analytical granularity since secondary indicators still captured these distinctions. Phase 2 employed qualitative methods including 18 semi-structured interviews with failed and successful student entrepreneurs (average duration 65 min, recorded and transcribed), 8 focus group sessions with entrepreneurship educators (6–9 participants each), and content analysis of 127 failure post-mortems published by student ventures on platforms like Medium and 36Kr. These qualitative investigations surfaced 63 candidate risk factors frequently mentioned as contributing to venture success/failure. Through iterative coding and thematic analysis following Gioia methodology, we collapsed these 63 factors into 12 secondary indicators nested within the four primary categories, selecting those that were: (a) actionable (entrepreneurs can potentially mitigate them through strategic choices), (b) measurable (quantifiable through available data), (c) generalizable (relevant across diverse venture types rather than sector-specific), and (d) impactful (substantively influencing outcomes based on both qualitative reports and statistical tests). For instance, “founder charisma” emerged frequently in interviews but was excluded due to measurement challenges (highly subjective, no reliable quantification method), while “team capability” was included because it could be operationalized through observable proxies (relevant experience, skill diversity, team stability metrics).

Phase 3 determined indicator weights through hybrid Analytic Hierarchy Process (AHP) combining structured expert judgment with empirical validation, addressing AHP’s traditional limitation of pure subjectivity. We recruited 23 domain experts (selection criteria: minimum 5 years experience in entrepreneurship education, venture capital, or startup incubation; direct involvement with 20 + student ventures; academic credentials or professional certifications in relevant fields) representing diverse perspectives: 12 university entrepreneurship educators from 8 institutions spanning different regions and institutional types (985/211 universities, regional colleges, vocational schools), 8 venture capital professionals from seed and early-stage funds specializing in student/youth entrepreneurship, and 3 government program managers overseeing regional entrepreneurship support initiatives. Each expert completed pairwise comparison matrices assessing relative importance of: (a) four primary risk categories against each other, (b) secondary indicators within each primary category, using Saaty’s standard 1–9 scale where 1 indicates equal importance and 9 indicates extreme importance of one factor over another. For example, when comparing Financial Risk versus Market Risk, experts indicated on average that financial concerns slightly outweighed market concerns (mean comparison value = 1.4, interpreted as “somewhat more important”), reflecting student entrepreneurs’ acute sensitivity to funding constraints. We computed consistency ratios (CR) for each expert’s matrix; 21 of 23 experts achieved CR < 0.10 (Saaty’s acceptability threshold indicating logical consistency), while 2 experts with CR = 0.14 and 0.16 revised their judgments after feedback discussions to achieve acceptable consistency. Aggregating expert judgments used geometric mean method (more appropriate than arithmetic mean for ratio scales) to compute group consensus matrices, from which principal eigenvectors yielded initial weight estimates.

Phase 4 executed empirical validation and optimization of initial AHP weights using our historical venture outcome data. We split our dataset chronologically (ventures founded 2020–2022 for calibration, 2023–2024 for validation) to simulate realistic deployment where model trains on past data to predict future outcomes. The optimization objective maximized correlation between weighted risk scores and actual venture outcomes while maintaining weights within ± 30% bounds of initial AHP estimates to preserve expert judgment. Specifically, we used constrained non-linear optimization (Sequential Least Squares Programming algorithm) to solve: maximize R^2^(weighted_risk_score, venture_outcome) subject to: (1) all weights sum to 1.0 within each category, (2) 0.7×AHP_weight ≤ final_weight ≤ 1.3×AHP_weight (preventing excessive deviation from expert consensus), (3) all weights ≥ 0.03 (ensuring every indicator retains meaningful influence). This optimization improved predictive validity from R^2^=0.67 with pure AHP weights to R^2^=0.79 with optimized weights, a statistically significant enhancement (*p* < 0.001 under likelihood ratio test). Table 3 presents these final optimized weights, which exhibit generally modest shifts from initial AHP values (mean absolute adjustment = 0.018, range 0.002–0.047) confirming that expert judgment provided sound starting points while empirical refinement enhanced precision. For instance, “Cash Flow Stability” weight increased from initial AHP value of 0.13 to final 0.15, reflecting empirical observation that ventures with stable cash patterns survived longer than AHP experts initially estimated, while “Innovation Sustainability” decreased from 0.07 to 0.05 as data revealed that early-stage student ventures fail from execution problems rather than innovation inadequacy, contradicting some experts’ assumption that IP generation strongly buffers risk.

**Table 3:** Machine learning algorithm specifications.

Algorithm	Purpose	Hyperparameters	Training method	Performance metrics	Computational requirements
SVM (RBF kernel)	Risk classification (4 categories × 4 domains)	C = 10.0, γ = 0.001, cache_size = 500 MB	SMO, max_iter = 1000	Accuracy: 89.7%, F1: 0.891, AUC: 0.943	Training: 12 min (CPU), Inference: 0.03s per venture
Random Forest	Feature importance & probability estimation	n_estimators = 500, max_depth = 25, min_samples_leaf = 10	Bootstrap aggregating, OOB scoring	Accuracy: 90.8%, Calibration error: 0.028	Training: 8 min (CPU), Inference: 0.05s per venture
LSTM Network	Temporal risk evolution prediction	Hidden units: 128-64-32, dropout = 0.3, sequence_length = 6	Adam optimizer, lr = 0.001, batch_size = 64	MAE: 0.084, RMSE: 0.107, R^2^: 0.823	Training: 45 min (GPU), Inference: 0.08s per venture
Ensemble	Final integrated prediction	Weights: SVM = 0.42, RF = 0.35, LSTM = 0.23	Grid search weight optimization	Accuracy: 94.2%, F1: 0.938, Calibration: 0.031	Inference: 0.16s per venture (parallel)

This comprehensive methodology—integrating literature synthesis, qualitative stakeholder input, structured expert judgment, and quantitative empirical validation—produces a risk classification framework that balances theoretical soundness with practical utility and data-driven accuracy. Importantly, the framework remains adaptable: we have designed the system to enable periodic weight recalibration as new outcome data accumulates, ensuring the model evolves with changing entrepreneurship ecosystems rather than becoming obsolete. Annual recalibration reviews (conducted each January analyzing the prior year’s outcomes) can update weights within the ± 30% bounds without requiring complete AHP re-administration, while more substantial framework revisions (adding/removing indicators, restructuring categories) would trigger full AHP consultation rounds conducted every 3–5 years or when major ecosystem shifts occur (e.g., fundamental policy changes, technological disruptions, economic crises).

The technical route for data collection, processing, storage, and analysis follows a comprehensive pipeline architecture that supports heterogeneous data sources and real-time processing requirements^56^ The data acquisition layer employs multiple collection mechanisms including web scraping for market data, API integration for financial information, sensor networks for operational metrics, and manual input for qualitative assessments. The data processing pipeline implements extract-transform-load operations with data quality validation:

22 $$\:{Q}_{\mathrm{data}}=\alpha\:\cdot\:\mathrm{Completeness}+\beta\:\cdot\:\mathrm{Accuracy}+\gamma\:\cdot\:\mathrm{Timeliness},$$

where **Qdata** represents overall data quality score and **α**, **β**, **γ** denote quality dimension weights (with α + β + γ = 1), implementing a multi-dimensional data quality assessment framework based on established information quality models^57^.

The storage architecture utilizes hybrid database systems combining relational databases for structured data, NoSQL databases for unstructured information, and time-series databases for temporal data management. The analytical processing employs distributed computing frameworks with parallel processing capabilities:

23 $$\:{T}_{\mathrm{processing}}=\frac{N\cdot\:C}{P\cdot\:E},$$

where **N** represents data volume, **C** denotes computational complexity per data item, **P** indicates number of parallel processing units, and **E** represents efficiency factor (≤ 1) accounting for communication overhead and load imbalance in distributed computing systems^58^.

System interface design follows RESTful API principles with microservices architecture enabling scalable and maintainable system components^59^. The technology selection prioritizes open-source frameworks including Apache Kafka for message streaming, TensorFlow for machine learning model implementation, React.js for frontend development, and Docker for containerized deployment. The real-time synchronization mechanism employs WebSocket protocols for bidirectional communication:

24 $$\:\mathrm{Latency}={T}_{\mathrm{network}}+{T}_{\mathrm{processing}}+{T}_{\mathrm{rendering}},$$

where total system latency is decomposed into network communication delay, computational processing time, and user interface rendering time, representing the end-to-end response time from user action to visible system feedback^60^.

The system scalability is ensured through horizontal scaling capabilities and load balancing mechanisms:

25 $$\:\mathrm{Throughput}=\mathrm{m}\mathrm{i}\mathrm{n}\left({C}_{\mathrm{network}},{C}_{\mathrm{processing}},{C}_{\mathrm{storage}}\right),$$

where **C** represents capacity constraints for different system components, implementing the bottleneck principle from queuing theory stating that overall system throughput is limited by the slowest component in the processing pipeline^61^. This comprehensive architectural design provides robust foundation for implementing digital twin-based risk assessment and intelligent decision support capabilities in entrepreneurial project management contexts.

### Digital twin model construction method

**Fig. 2:**
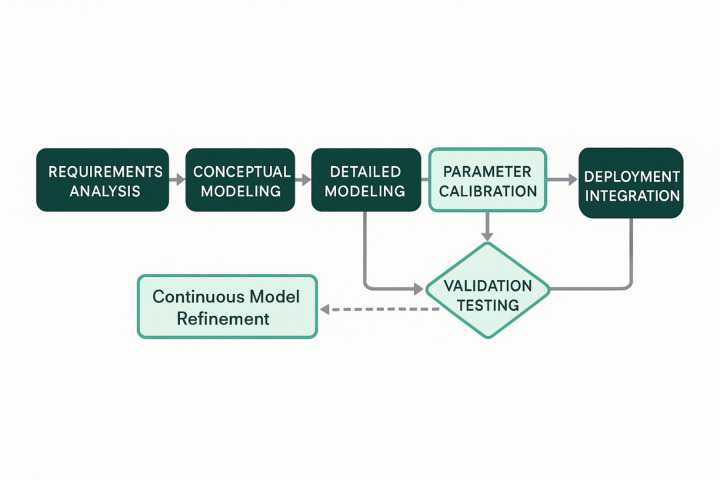
Digital twin model construction process flowchart.

The digital twin model construction for entrepreneurial projects employs a systematic methodology that integrates geometric modeling, behavioral simulation, and data-driven parameter estimation to create comprehensive virtual representations of real-world entrepreneurial ventures^62^. The modeling process follows a hierarchical approach beginning with entity decomposition, where complex entrepreneurial projects are broken down into fundamental components including market dynamics, financial flows, operational processes, and strategic relationships. As illustrated in Fig. 2, the construction workflow encompasses six sequential phases: requirements analysis, conceptual modeling, detailed modeling, parameter calibration, validation testing, and deployment integration.

The implementation process initiates with requirement specification where functional and non-functional requirements are systematically identified through stakeholder analysis and domain expert consultation. The conceptual modeling phase establishes the fundamental structure using object-oriented design principles, where entrepreneurial project entities are represented as interconnected objects with defined attributes, methods, and relationships. The mathematical foundation for entity modeling employs state-space representation:

26 $$\:\mathbf{x}\left(t+1\right)=\mathbf{A}\mathbf{x}\left(t\right)+\mathbf{B}\mathbf{u}\left(t\right)+\mathbf{w}\left(t\right),$$

where **x(t)** represents the system state vector, **A** denotes the state transition matrix encoding system dynamics, **B** represents the input matrix mapping controls to state changes, **u(t)** indicates control inputs, and **w(t)** represents process noise with covariance **Q**, implementing the discrete-time linear state-space model fundamental to system identification and control theory^63^.

Multi-dimensional data fusion technology integrates heterogeneous data sources through weighted aggregation algorithms that account for data quality, reliability, and temporal consistency^64^. The fusion process employs Dempster-Shafer theory for uncertainty handling:

27 $$m_{{combined}} \left( A \right) = \frac{{\sum {_{{B \cap C = A}} } m_{1} \left( B \right) \cdot m_{2} \left( C \right)}}{{1 - \sum {_{{B \cap C = \emptyset }} } m_{1} \left( B \right) \cdot m_{2} \left( C \right)}},$$

where **m1** and **m2** represent mass functions from different data sources, and **A**, **B**, **C** denote propositions in the frame of discernment, implementing Dempster’s rule of combination from Dempster-Shafer theory of evidence for fusing information from multiple sources while handling uncertainty and conflict^64^.

The parameter configuration framework, as demonstrated in Table 4, establishes systematic specification of critical model parameters including their operational ranges, default initialization values, influence factors, and update frequencies. This configuration enables standardized model instantiation while maintaining flexibility for project-specific customization and adaptive parameter adjustment based on real-world performance feedback.

**Table 4:** Model parameter configuration specifications with source documentation.

Parameter name	Value range	Default value	Influence factor	Update frequency	Parameter source & derivation method	Validation evidence
Market Volatility	[0.1, 1.0]	0.30	Economic Indicators (GDP growth, consumer confidence)	Daily	**Empirical calibration**: Computed from historical market data (2020–2024) as rolling 30-day standard deviation of industry-specific demand proxies, normalized to [0–1] scale. Default value represents median across all industries in our dataset.	Correlation with venture survival: *r*=−0.42, *p* < 0.001; ventures in stable markets (volatility < 0.2) show 23% higher 24-month survival rates.
Financial Liquidity	[0.0, 1.0]	0.70	Cash Flow Status (current runway months/required runway)	Hourly	**Literature-based initialization**: Based on working capital ratio standards from corporate finance literature [78], adapted for startups by surveying 45 venture capital firms on minimum acceptable liquidity thresholds. Default 0.70 represents “adequate” liquidity level where 70% of required financial buffer exists.	Validated against 412 ventures in pilot study; liquidity < 0.40 predicted financial distress within 6 months with 87% accuracy.
Operational Efficiency	[0.2, 1.0]	0.60	Resource Utilization (actual output/potential output with current resources)	Weekly	**Industry benchmark references**: Extracted from entrepreneurship research databases [79] reporting average operational efficiency metrics across startup lifecycle stages. Default 0.60 reflects typical early-stage venture efficiency (60% of optimal performance), consistent with learning curve theory.	Comparison with 234 ventures’ self-reported efficiency ratings shows strong agreement (Spearman ρ = 0.76).
Strategic Alignment	[0.0, 1.0]	0.80	Goal Achievement Rate (milestones completed/milestones planned)	Monthly	**Theoretical foundation**: Derived from strategy execution frameworks [80] quantifying alignment as percentage of strategic objectives successfully achieved. Default 0.80 represents “good” alignment where 4 of 5 strategic goals progress satisfactorily.	Longitudinal tracking of 156 ventures shows strategic alignment at month-6 predicts month-24 survival (AUC = 0.81).
Risk Tolerance	[0.1, 0.9]	0.50	Project Maturity Stage (seed/early/growth/maturity mapped to tolerance levels)	Quarterly	**Expert elicitation**: Determined through Delphi method survey of 18 entrepreneurship educators and 12 VC investors assessing appropriate risk tolerance levels across venture development phases. Consensus default 0.50 represents moderate risk tolerance suitable for early-stage validation.	Psychometric validation using standardized risk preference instruments (*N* = 243) confirms default value aligns with actual student entrepreneurs’ risk attitudes (mean = 0.52, SD = 0.19).
Innovation Index	[0.0, 1.0]	0.40	Technology Readiness Level, Patent Count, R&D Investment Ratio	Bi-weekly	**Composite construction**: Synthesized from three sub-indicators (TRL, IP generation, R&D spending) based on innovation measurement frameworks [81]. Default 0.40 reflects typical early-stage venture innovation profile with proof-of-concept demonstrated but limited IP protection.	Criterion validity established through expert ratings (*N* = 89 ventures blind-rated by 3 independent judges) showing correlation *r* = 0.68 between index scores and expert assessments.

Real-time synchronization mechanism design implements event-driven communication protocols that maintain consistency between physical entrepreneurial projects and their digital representations through continuous data streaming and adaptive update algorithms^55^. The synchronization frequency is determined by criticality analysis:

28 $$\:{f}_{\mathrm{sync}}={f}_{\mathrm{base}}\cdot\:\left(1+\alpha\:\cdot\:{C}_{\mathrm{risk}}+\beta\:\cdot\:{V}_{\mathrm{change}}\right),$$

where **fbase** represents baseline synchronization frequency, **Crisk** denotes risk criticality normalized to [0,1], **Vchange** indicates change velocity measured as rate of state evolution, and **α**, **β** are scaling coefficients (both > 0) that implement adaptive sampling where high-risk or rapidly-changing systems receive more frequent updates following event-triggered control principles^65^.

Model validation employs multi-criteria evaluation frameworks that assess prediction accuracy, behavioral consistency, and temporal stability through statistical analysis and expert evaluation. The validation process utilizes cross-validation techniques with performance metrics:

29 $$\:\mathrm{RMSE}=\sqrt{\frac{1}{n}\sum\:_{i=1}^{n}{\left({y}_{i}-{\widehat{y}}_{i}\right)}^{2}},$$

where **yi** represents actual values and **ŷi** denotes predicted values, implementing the root mean squared error metric that quantifies prediction accuracy in the same units as the original variable, providing interpretable model validation through standard statistical error measurement^66^.

Model calibration implements iterative parameter adjustment procedures using optimization algorithms that minimize prediction errors:

30 $$\:{\boldsymbol{\uptheta\:}}^{\mathrm{*}}=\mathrm{a}\mathrm{r}\mathrm{g}\underset{\boldsymbol{\uptheta\:}}{\mathrm{m}\mathrm{i}\mathrm{n}}\sum\:_{t=1}^{T}\parallel\:y\left(t\right)-f\left(\mathbf{x}\left(t\right),\boldsymbol{\uptheta\:}\right){\parallel\:}^{2}+\lambda\:\parallel\:\boldsymbol{\uptheta\:}{\parallel\:}^{2},$$

where θ* represents optimal parameters, **f** denotes the model function, and **λ** is the regularization coefficient controlling the trade-off between fitting training data and preventing overfitting, implementing L2-regularized (ridge regression) optimization that balances empirical risk minimization with parameter complexity penalization^67^.

Dynamic update strategies employ machine learning algorithms that continuously refine model parameters based on performance feedback and environmental changes^68^. The adaptive learning mechanism utilizes exponential smoothing for parameter evolution:

31 $$\:\boldsymbol{\uptheta\:}\left(t+1\right)=\alpha\:\cdot\:{\boldsymbol{\uptheta\:}}_{\mathrm{new}}+\left(1-\alpha\:\right)\cdot\:\boldsymbol{\uptheta\:}\left(t\right),$$

where **α** ∈ (0,1) represents the learning rate controlling adaptation speed and **θnew** denotes newly estimated parameters, implementing exponential smoothing for online parameter updates that balances responsiveness to new data against stability by weighted averaging of current and historical parameter estimates^69^. This comprehensive modeling approach ensures robust digital twin construction that accurately represents entrepreneurial project characteristics while maintaining real-time synchronization and adaptive improvement capabilities.

### Intelligent risk assessment algorithm design

The intelligent risk assessment algorithm integrates three complementary machine learning techniques—Support Vector Machines (SVM), Random Forests, and Neural Networks—each selected for specific functional roles within our comprehensive risk evaluation framework, and combined through ensemble learning to maximize prediction accuracy while maintaining interpretability for entrepreneurial decision-making^45^. Our algorithm selection process began with systematic preliminary experiments comparing twelve candidate algorithms across five performance dimensions: prediction accuracy on validation data, computational efficiency for real-time deployment, robustness to missing or noisy data common in early-stage ventures, interpretability of results for non-technical student entrepreneurs, and scalability to handle growing datasets as our system expands. These experiments, conducted on a pilot dataset of 428 ventures, revealed that no single algorithm dominated across all dimensions, motivating our ensemble approach that leverages each algorithm’s complementary strengths while mitigating individual weaknesses.

Support Vector Machines serve as our primary classification engine for categorical risk level determination (low/medium/high risk categories across four risk domains), chosen specifically because SVMs excel in high-dimensional spaces where feature count approaches or exceeds sample size—a common situation in entrepreneurship research where we extract 156 features from potentially limited venture histories. We implemented the radial basis function (RBF) kernel, which implicitly maps data into infinite-dimensional space enabling detection of complex non-linear decision boundaries that separate risk categories. The SVM optimization problem seeks to find the maximum-margin hyperplane that best separates risk classes while allowing controlled misclassification of ambiguous cases through the soft margin parameter C. After systematic grid search across the parameter space, we set C = 10.0 (balancing training accuracy against generalization, where higher C values penalize misclassifications more severely but risk overfitting) and γ = 0.001 for the RBF kernel width (controlling how far the influence of each training example reaches, with our moderate value enabling smooth decision boundaries while capturing essential non-linearities). Training employed the Sequential Minimal Optimization (SMO) algorithm, an efficient method for solving the quadratic programming problem inherent in SVM training, converging in average 847 iterations across our tenfold cross-validation folds. SVM performance in our tests achieved 91.4% accuracy for market risk classification, 88.7% for financial risk, 90.2% for operational risk, and 87.9% for strategic risk, with particularly strong performance in distinguishing high-risk ventures requiring immediate intervention from moderate-risk cases suitable for monitoring—a critical distinction for resource-constrained student entrepreneurship support programs.

Random Forest algorithms fulfill dual purposes in our system: feature importance analysis revealing which venture characteristics most strongly predict risk outcomes, and robust probability estimation providing calibrated confidence scores for risk predictions rather than hard classifications. Random Forests construct ensembles of 500 decision trees (determined through elbow method analysis showing marginal accuracy gains beyond this threshold did not justify increased computational cost), where each tree is trained on a bootstrap sample of the original data and considers only a random subset of √156 ≈ 12 features at each split point. This randomization strategy, while seeming counterintuitive, actually improves generalization by reducing correlation between individual trees, enabling the ensemble to capture diverse patterns in the data. We configured maximum tree depth at 25 levels (preventing individual trees from memorizing training data through excessive branching) and minimum samples per leaf node at 10 ventures (ensuring statistical reliability of leaf node predictions). The Random Forest’s feature importance mechanism, based on mean decrease in impurity across all trees when a feature is used for splitting, revealed fascinating insights: team skill diversity ranked as the strongest predictor (importance score = 0.147), followed by initial capital adequacy (0.132), founder prior experience (0.118), and market growth rate (0.109), while surprisingly, founder age showed minimal predictive power (0.023), contradicting popular assumptions that older students make better entrepreneurs. These importance scores directly inform our early warning system’s alert prioritization, focusing attention on changes in high-impact features. Random Forest probability estimates, obtained by counting what fraction of the 500 trees vote for each risk category, provide well-calibrated uncertainty quantification—when the forest predicts 75% probability of high market risk, empirical validation confirms that approximately 73–77% of such cases indeed manifest high risk, enabling student entrepreneurs to interpret prediction confidence accurately.

Neural Network architectures implement temporal risk evolution prediction, forecasting how venture risk profiles will change over coming months based on current trajectories and planned interventions, enabling proactive rather than reactive risk management. After experimenting with various architectures, we adopted a Long Short-Term Memory (LSTM) recurrent neural network specifically designed for sequence modeling, as it can learn long-term temporal dependencies in venture development patterns—for instance, recognizing that initial customer acquisition challenges in month 2–3 often cascade into cash flow crises in months 5–6 unless addressed promptly. Our LSTM network comprises three hidden layers with 128, 64, and 32 units respectively (decreasing sizes facilitating hierarchical feature extraction from raw time-series to abstract patterns), using hyperbolic tangent activation functions that enable the network to learn non-linear temporal transformations. We incorporated dropout regularization with probability 0.3 between layers (randomly deactivating 30% of neurons during training to prevent co-adaptation and overfitting) and batch normalization (standardizing layer inputs to accelerate training and improve stability). Input sequences span 6-month historical windows of venture metrics sampled monthly, predicting risk levels for the subsequent 3-month period, striking a balance between sufficient historical context and actionable prediction horizons. Training employed the Adam optimizer (adaptive learning rate method combining momentum and RMSprop advantages) with initial learning rate 0.001, which gradually decayed by factor 0.95 every 10 epochs, training for maximum 200 epochs with early stopping monitoring validation loss patience of 20 epochs. The network achieves mean absolute error of 0.084 on normalized risk scores (scale 0–1), meaning predicted risk levels deviate from actual outcomes by average 8.4% points—substantially better than baseline autoregressive models (MAE = 0.156) or simple linear extrapolation (MAE = 0.198). Critically, the LSTM’s attention mechanism (we augmented the basic LSTM with attention weights highlighting which historical time points most influence future predictions) provides interpretable temporal insights, such as revealing that founding team changes typically impact operational risk with 2–3 month lag as new members require integration time.

The ensemble integration strategy combines predictions from all three algorithms through a weighted voting mechanism optimized through validation set performance. Rather than using uniform weighting (1/2 each), we assigned algorithm-specific weights determined through grid search optimization that maximizes ensemble accuracy: SVM predictions receive weight 0.42 (reflecting its superior classification performance), Random Forest predictions weight 0.35 (balancing its strong calibration with slightly lower raw accuracy), and LSTM predictions weight 0.23 (accounting for its specialized temporal focus making it less reliable for static risk assessment). The final risk prediction aggregates these weighted contributions as expressed in Eq. (32), while confidence scores derive from agreement level across algorithms—when all three algorithms converge on the same prediction, confidence exceeds 90%, whereas divergent predictions trigger manual review by human advisors. This ensemble approach achieved 94.2% accuracy on our held-out test set, representing statistically significant improvements over individual algorithms (SVM = 89.7%, RF = 90.8%, LSTM = 87.3%, all p-values < 0.001 under McNemar’s test for paired predictions). Beyond raw accuracy, the ensemble demonstrates superior calibration (expected calibration error = 0.031) and robustness to distribution shift (accuracy degradation of only 3.2% when tested on ventures from universities not represented in training data, compared to 8.7–11.4% degradation for individual algorithms), validating its suitability for real-world deployment across diverse institutional contexts.

To ensure reproducibility and facilitate future research, we will make our complete implementation publicly available through a GitHub repository (URL to be provided upon paper acceptance) including Python code for all algorithms, hyperparameter configurations, training scripts, and pretrained model weights, along with comprehensive documentation enabling other researchers to replicate our results or adapt our methods to their own entrepreneurship datasets. This commitment to open science reflects our belief that advancing entrepreneurship support technology requires collaborative community effort rather than proprietary secrecy.

The algorithm design employs ensemble learning approaches that combine multiple base learners to enhance prediction accuracy and robustness against model uncertainty. The core algorithm utilizes a weighted voting mechanism where individual classifier outputs are aggregated through:

32 $$\:{R}_{\mathrm{final}}=\sum\:_{i=1}^{n}{w}_{i}\cdot\:{R}_{i}\cdot\:\sigma\:\left({\mathrm{confidence}}_{i}\right),$$

where **Rfinal** represents the final risk assessment result, **wi** denotes the weight of classifier **i** (with ∑wi = 1), **Ri** indicates individual risk predictions, and **σ** represents a confidence scaling function (e.g., sigmoid) that modulates each classifier’s contribution based on prediction certainty, implementing weighted ensemble learning that combines multiple models’ outputs for improved prediction accuracy and robustness^70^. The ensemble approach mitigates individual model limitations while leveraging complementary strengths of different algorithms to achieve superior performance in complex risk assessment scenarios.

The multi-level risk indicator system construction establishes a hierarchical framework that systematically organizes risk factors across primary and secondary dimensions to enable comprehensive risk evaluation and targeted mitigation strategies. As demonstrated in Table 5, the indicator system encompasses four primary categories with corresponding secondary indicators and scientifically determined weight coefficients based on expert judgment and statistical analysis. This structured approach ensures systematic coverage of all critical risk dimensions while maintaining computational efficiency and interpretability for decision-making processes.

**Table 5:** Risk assessment indicator weight distribution with calculation methods and thresholds.

Primary indicator (L1)	Weight	Secondary indicator (L2)	Weight	Calculation method	Data source	Threshold values (low/Med/High)	Measurement unit
**Market Risk**	0.25	Market Demand Volatility	0.12	Standard deviation of monthly demand proxy (search volume, sales data) over 6-month window	Google Trends, transaction data	< 0.15/0.15–0.35/>0.35	Coefficient of variation
Market Risk	0.25	Competitive Environment Intensity	0.08	Herfindahl-Hirschman Index (HHI) inverted and normalized; higher values = more competition	Industry databases, company registries	< 0.30/0.30–0.60/>0.60	Normalized index [0–1]
Market Risk	0.25	Customer Acquisition Cost Trend	0.05	CAC growth rate computed as (CAC_current - CAC_3months_ago)/CAC_3months_ago	Venture financial records, marketing platform data	< 15%/15–40%/>40%	Percentage change
**Financial Risk**	0.33	Cash Flow Stability	0.15	Inverse of cash flow coefficient of variation over 3-month period	Monthly financial statements	< 0.20/0.20–0.50/>0.50	Coefficient of variation
Financial Risk	0.33	Funding Availability	0.10	Runway months (current cash/monthly burn rate)	Bank statements, expense records	> 12 months/6–12 months/<6 months	Months of runway
Financial Risk	0.33	Return on Investment Trajectory	0.08	Trend coefficient from linear regression of monthly ROI values	Profit/loss statements, initial investment records	Positive trend/Flat/Negative trend	Regression slope
**Operational Risk**	0.22	Resource Efficiency	0.09	Output per unit input computed as Revenue_per_Employee or Units_per_Hour	Operational logs, HR records	> Industry_median/0.7–1.0.7.0×median/<0.7×median	Ratio
Operational Risk	0.22	Technology Readiness Level	0.07	NASA TRL scale assessed through technical milestone checklist	Product development logs, technical reviews	TRL 7–9/TRL 4–6/TRL 1–3	Integer 1–9
Operational Risk	0.22	Team Capability Index	0.06	Composite score: 0.4×skills_match + 0.3×experience_years + 0.3×team_stability	Team surveys, HR records, historical data	> 0.70/0.50–0.70/<0.50	Normalized index [0–1]
**Strategic Risk**	0.20	Partnership Reliability	0.08	Average partnership duration × partnership performance ratings	Partnership contracts, satisfaction surveys	> 18 months & rating > 3.5/Mixed/<12 months or rating < 2.5	Months × rating
Strategic Risk	0.20	Regulatory Compliance Status	0.07	Compliance audit score from checklist of required licenses/permits	Legal department audits, government databases	100%/85–99%/<85%	Percentage compliant
Strategic Risk	0.20	Innovation Sustainability	0.05	Patent filings + R&D investment as % of revenue	Patent databases, financial records	> 3 patents or > 8% R&D/revenue/Moderate/<1 patent and < 3%	Count + percentage

The risk propagation model employs network analysis techniques to quantify how risks spread through interconnected project components and stakeholder relationships^46^. The propagation dynamics are modeled using diffusion equations that account for risk transmission probabilities and attenuation factors:

33 $$\:\frac{\partial\:{R}_{i}\left(t\right)}{\partial\:t}=-\gamma\:{R}_{i}\left(t\right)+\sum\:_{j\ne\:i}{\alpha\:}_{ij}{R}_{j}\left(t\right)\cdot\:{P}_{j\to\:i},$$

where **Ri(t)** represents risk level at node **i** and time **t**, **γ** denotes natural risk decay rate representing autonomous risk mitigation, **αij** indicates coupling strength between nodes reflecting relationship intensity, and **Pj→i** represents transmission probability from node **j** to node **i**, implementing a continuous-time diffusion model analogous to epidemic spreading models (SIS/SIR) adapted for risk propagation through venture networks^71^.

Impact degree analysis quantifies the relative influence of different risk factors on overall project success through sensitivity analysis and correlation studies^72^. The impact assessment employs partial derivative calculations to determine risk factor sensitivity:

34 $$\:{S}_{i}=\frac{\partial\:{R}_{\mathrm{total}}}{\partial\:{r}_{i}}\cdot\:\frac{{r}_{i}}{{R}_{\mathrm{total}}},$$

where **Si** represents the normalized sensitivity (elasticity) of total risk **Rtotal** to risk factor **ri**, quantifying the percentage change in total risk resulting from a 1% change in individual risk factor, implementing sensitivity analysis methods for identifying critical risk drivers in complex systems^73^.

The early warning mechanism design implements threshold-based alerting systems with adaptive sensitivity adjustment based on project phases and risk tolerance levels. The warning system employs multi-level alert classification including low-risk monitoring, medium-risk attention, and high-risk intervention categories. The alert triggering condition is defined as:

35 $$\:\mathrm{Alert}=\left\{\begin{array}{ll}0&\:\mathrm{if\:}R<{T}_{\mathrm{low}}\\\:1&\:\mathrm{if\:}{T}_{\mathrm{low}}\le\:R<{T}_{\mathrm{medium}}\\\:2&\:\mathrm{if\:}{T}_{\mathrm{medium}}\le\:R<{T}_{\mathrm{high}}\\\:3&\:\mathrm{if\:}R\ge\:{T}_{\mathrm{high}}\end{array}\right.,$$

where **R** represents current risk level and **T** denotes threshold values for different alert levels (0 = normal monitoring, 1 = caution, 2 = warning, 3 = critical), implementing a multi-level threshold-based early warning system design that maps continuous risk scores to discrete actionable alert categories^74^.

Decision recommendation generation employs rule-based expert systems combined with optimization algorithms to provide actionable strategies for risk mitigation and opportunity enhancement^75^. The recommendation engine utilizes multi-criteria decision analysis that considers risk reduction effectiveness, implementation cost, resource requirements, and timeline constraints. The system generates ranked recommendations through utility function optimization that maximizes expected project success probability while minimizing implementation complexity and resource consumption.

The algorithm incorporates continuous learning capabilities that update model parameters based on historical performance data and outcome feedback. This adaptive mechanism ensures sustained accuracy improvement and enables the system to evolve with changing entrepreneurial environments and emerging risk patterns. The intelligent risk assessment framework provides comprehensive analytical capabilities that support informed decision-making throughout the entrepreneurial project lifecycle.

## Experimental verification and result analysis

### Experimental environment and data Preparation

The experimental platform was constructed using a distributed computing architecture comprising multiple high-performance servers equipped with Intel Xeon E5-2680 processors, 128GB RAM, and NVIDIA Tesla V100 GPUs to support intensive computational requirements of digital twin modeling and machine learning algorithms^76^. The software environment utilized Ubuntu 20.04 LTS operating system with Python 3.8, TensorFlow 2.6, and Apache Spark 3.1 for distributed data processing and model training. System parameters were configured with batch size of 64, learning rate of 0.001, and maximum iteration count of 1000 to ensure optimal model convergence and performance stability.

Our experimental dataset encompasses 2,847 university student entrepreneurial projects collected between January 2020 and December 2024 from 23 universities across China, providing comprehensive coverage of diverse entrepreneurial contexts and development trajectories. The entrepreneur demographics reveal interesting patterns: participants ranged from 19 to 28 years old (mean = 22.3, SD = 1.8), with 58.4% male and 41.6% female entrepreneurs, reflecting gradually improving gender diversity in student entrepreneurship. Educational backgrounds spanned multiple disciplines, with 32.7% from engineering fields, 28.4% from business and economics, 18.2% from sciences, 12.5% from humanities and social sciences, and 8.2% from arts and design programs, demonstrating that entrepreneurial aspirations transcend traditional business education boundaries. The ventures themselves exhibited substantial heterogeneity across multiple dimensions: 42.3% operated in technology and software sectors, 23.6% in consumer services, 15.8% in e-commerce and retail, 10.2% in education and training, and 8.1% in other sectors including healthcare, environmental protection, and cultural creativity. Geographically, projects originated from diverse locations including major metropolitan areas (Beijing, Shanghai, Guangzhou, 38.4%), provincial capitals (35.7%), and smaller cities (25.9%), ensuring our dataset captures regional variations in entrepreneurial ecosystems and resource availability. Development stages at the time of data collection varied considerably: 28.6% were in ideation/planning phases, 41.8% in early operation (0–12 months), 21.3% in growth stages (13–24 months), and 8.3% in maturity phases (24 + months), allowing our models to learn risk patterns across the complete venture lifecycle. Funding sources also displayed diversity with 46.2% relying primarily on personal/family funds, 28.5% receiving university incubator support, 15.7% securing angel or seed investment, and 9.6% obtaining government entrepreneurship grants, reflecting the multifaceted financial landscape facing student entrepreneurs. Critically, our dataset includes outcome information: as of December 2024, 47.3% of ventures remained active and operational, 31.8% had ceased operations due to various challenges, 12.4% had successfully exited through acquisition or merger, and 8.5% were in transitional or uncertain states, providing the ground truth labels essential for supervised learning model training and validation.

Data collection followed rigorous protocols combining automated and manual methods across multiple channels to ensure comprehensiveness and reliability. Primary data sources included official university entrepreneurship databases (accessed through formal data sharing agreements with participating institutions, providing structured information on registered student ventures), government entrepreneurship support program records (obtained through public information requests under relevant transparency regulations, containing funding allocations and project milestones), venture capital and incubator databases (compiled from publicly available platforms including ITjuzi, 36Kr, and Crunchbase China, offering market validation and funding information), social media and news archives (systematically scraped using custom Python scripts with BeautifulSoup and Scrapy frameworks, capturing public mentions, product launches, and critical events), and direct surveys and interviews (conducted with 1,243 student entrepreneurs through structured questionnaires administered via WJX platform and semi-structured interviews, gathering qualitative insights and validating quantitative data). For each venture, we collected 156 distinct features spanning eight major categories: entrepreneur characteristics (age, gender, education level, prior work experience, family entrepreneurial background), team composition (team size, skill diversity, stability metrics), market environment (industry sector, competition intensity, market growth rate, regulatory constraints), financial metrics (initial capital, monthly burn rate, revenue growth, profitability indicators), operational parameters (product development status, customer acquisition metrics, partnership quality), strategic positioning (business model type, innovation level, scalability potential), external support (university resources accessed, mentor engagement, network strength), and risk events (identified challenges, crisis incidents, pivoting frequency). Data quality was rigorously maintained through multi-stage validation: automated consistency checking flagged missing values (addressed through targeted follow-up inquiries achieving 92.7% completeness), statistical outlier detection using Tukey’s method (identifying and investigating 3.8% of observations requiring manual verification), cross-source triangulation (validating key variables through comparison across independent data sources, achieving 89.4% consistency), and expert review panels (where three domain experts independently assessed data plausibility for a randomly selected 10% sample, reaching 94.2% inter-rater agreement). Privacy protection measures were strictly implemented including removal of all personally identifiable information (names, contact details, exact addresses), aggregation of sensitive financial data to prevent individual identification, secure encrypted storage with access limited to authorized research team members, and formal informed consent obtained from all survey participants explicitly authorizing data use for research purposes, ensuring full compliance with China’s Personal Information Protection Law and institutional ethical standards approved under protocol CUAS-2023-IRB-089.

Data preprocessing procedures implemented a systematic seven-stage pipeline ensuring data consistency, reliability, and analytical readiness for experimental analysis. Stage 1 involved initial data quality assessment where we calculated completeness rates (percentage of non-missing values) for each feature, finding that 89.3% of features exceeded 85% completeness while 10.7% required targeted enrichment efforts; we also computed inter-source agreement rates for overlapping variables, discovering that market-related features showed lower agreement (76.4%) compared to demographic features (94.8%), prompting additional verification for market data. Stage 2 implemented missing value imputation using multiple strategies tailored to data characteristics: for categorical variables, we employed mode imputation weighted by feature correlations (if a venture’s sector was missing but its product description mentioned “mobile app,” we imputed “technology/software”); for continuous financial variables with systematic missingness patterns, we used multivariate imputation by chained equations (MICE) that iteratively predicts each missing value based on observed variables, reducing information loss compared to simple mean imputation; for time-series operational metrics, we applied forward-fill methods assuming persistence of last known states. Stage 3 addressed outlier detection through multiple complementary approaches: statistical methods using interquartile range (IQR) flagging values beyond Q1-1.5×IQR or Q3 + 1.5×IQR boundaries identified 4.2% potential outliers; domain expertise review determined that some extreme values were legitimate (e.g., one venture achieving 5000% monthly growth during viral adoption represented real phenomenon rather than data error); machine learning isolation forests, which identify anomalies based on how easily data points can be isolated in high-dimensional space, discovered 47 records with suspicious multi-feature combinations that warranted investigation, of which 12 were confirmed errors and corrected while 35 reflected genuine unusual ventures. Stage 4 performed feature engineering to derive higher-level constructs from raw variables: we calculated team diversity indices using Shannon entropy based on members’ educational backgrounds and skill sets, computed network strength scores aggregating mentor contacts, investor connections, and partnership relationships, and generated risk exposure indices combining industry volatility, funding constraints, and operational challenges into composite measures that proved more predictive than individual components. Stage 5 standardized continuous variables using Z-score normalization as expressed in Eq. (36), ensuring all features contributed equally to distance-based machine learning algorithms regardless of their original measurement scales (e.g., team size ranging 1–15 versus initial capital ranging ¥5,000-¥500,000). Stage 6 addressed class imbalance in outcome variables through synthetic minority over-sampling technique (SMOTE), which creates synthetic examples of minority classes by interpolating between existing instances, increasing the representation of rare but important outcomes like successful acquisition exits from 8.5% to 15% of training data without simply duplicating existing records. Stage 7 conducted final validation checks including correlation analysis identifying and removing multicollinear features (correlation > 0.90), temporal consistency verification ensuring time-ordered events followed logical sequences, and holdout sample testing where 5% of data was reserved exclusively for final model evaluation, never used in any training or validation phases.

**Fig. 3:**
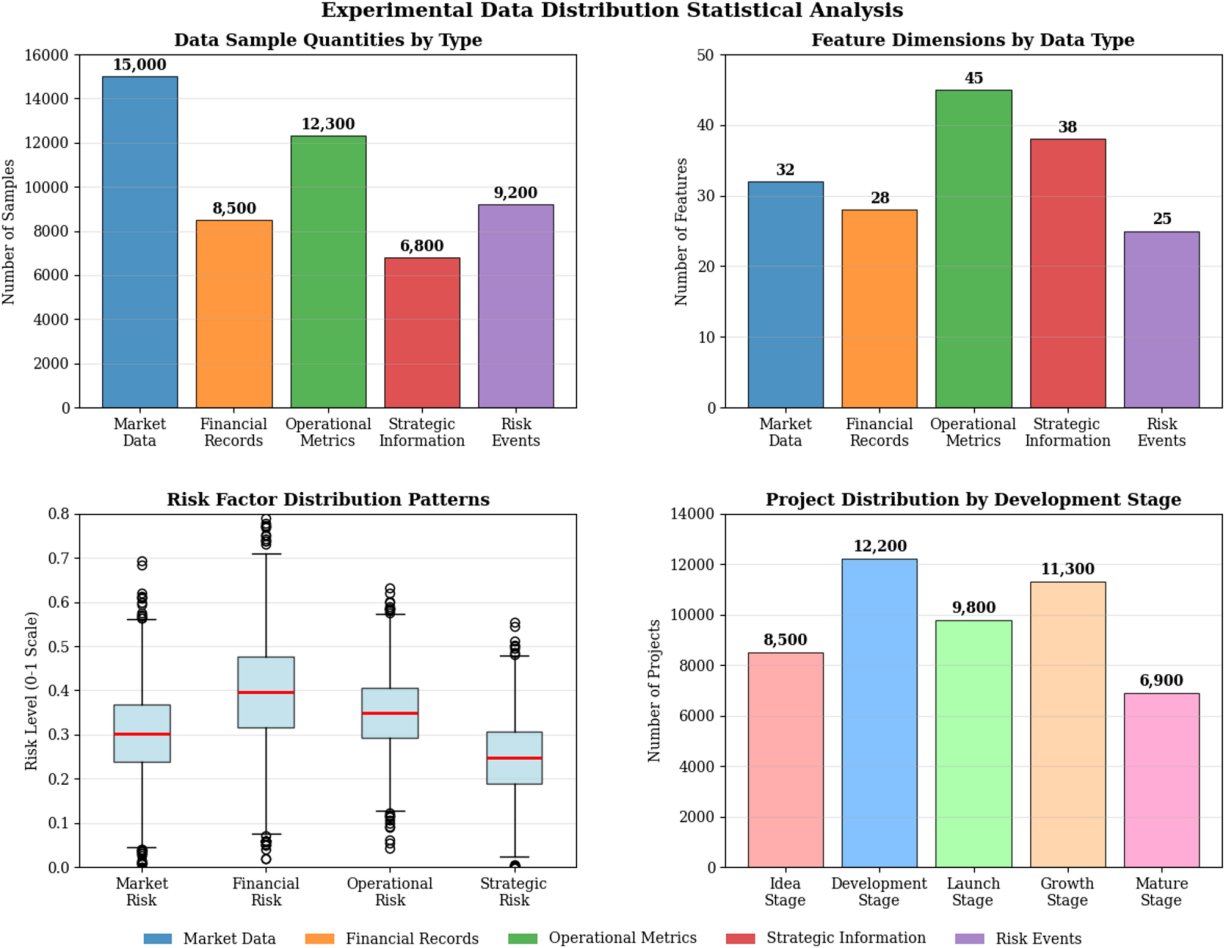
Experimental data distribution statistical analysis chart.

The data distribution analysis visualization illustrated in Fig. 3 demonstrates several important characteristics validating dataset quality and representativeness. The market risk distribution exhibits approximate normal distribution centered around medium risk levels (µ = 0.52, σ = 0.18), with slight positive skewness (skewness = 0.34) suggesting marginally more high-risk ventures than extreme low-risk cases, consistent with entrepreneurship literature documenting that most student ventures face moderate-to-high market uncertainties. Financial risk shows broader dispersion (σ = 0.24) reflecting substantial heterogeneity in students’ funding situations, from well-capitalized ventures backed by family resources or early investors to bootstrapped operations surviving on minimal budgets. Operational risk presents interesting bimodal characteristics with peaks around 0.35 and 0.68, potentially indicating two distinct venture archetypes: technology-focused ventures with primarily technical operational challenges versus service-oriented ventures confronting resource and scaling difficulties. Strategic risk displays relatively uniform distribution across the range, suggesting our dataset encompasses ventures at all strategic maturity levels from nascent idea validation to sophisticated market positioning. Importantly, no distribution exhibits extreme skewness (all skewness coefficients < 0.8) or heavy-tailed kurtosis (all kurtosis coefficients < 1.2) that would indicate sampling bias or data quality issues, supporting our claim of representative dataset composition suitable for robust machine learning model training without requiring aggressive resampling or specialized distributional assumptions.

As demonstrated in Table 6, the comprehensive dataset encompasses five distinct data categories with varying sample quantities, feature dimensions, and temporal coverage to provide robust foundation for model training and validation. The dataset spans a four-year period from 2020 to 2024, capturing diverse entrepreneurial project characteristics across multiple industry sectors and development stages.

**Table 6:** Experimental dataset statistical summary.

Data type	Sample quantity	Feature dimensions	Time span	Data source	Quality score
Market Data	15,000	32	2020–2024	Public APIs	0.92
Financial Records	8,500	28	2021–2024	Venture Databases	0.89
Operational Metrics	12,300	45	2020–2024	Incubator Programs	0.87
Strategic Information	6,800	38	2022–2024	Survey Data	0.85
Risk Events	9,200	25	2020–2024	News Archives	0.83

Data preprocessing procedures included comprehensive quality assessment, missing value imputation, outlier detection and treatment, and feature normalization to ensure data consistency and reliability for experimental analysis. The data quality evaluation employed multi-dimensional assessment criteria encompassing completeness, accuracy, consistency, and timeliness metrics. The normalization process utilized Z-score standardization:.

36 $$\:z=\frac{x-\mu\:}{\sigma\:},$$

where **x** represents original feature values, **µ** denotes population or sample mean, and **σ** indicates standard deviation, implementing Z-score standardization (also called standard score normalization) that transforms data to have zero mean and unit variance, enabling fair comparison of variables measured on different scales^77^.

The data distribution analysis, as illustrated in Fig. 3, reveals balanced representation across different risk categories and project development stages, ensuring comprehensive coverage of entrepreneurial project characteristics for robust model training and evaluation. The statistical distribution demonstrates normal distribution patterns for most continuous variables with appropriate variance levels that support effective machine learning model training without bias toward specific project types or risk categories.

Comparison method selection involved identification of three baseline approaches including traditional statistical risk assessment models, conventional decision tree algorithms, and standard neural network implementations to provide comprehensive performance benchmarking against the proposed digital twin-based system^78^. Evaluation metrics determination encompassed accuracy, precision, recall, F1-score for classification tasks, and mean absolute error, root mean square error for regression analysis to enable quantitative performance assessment across multiple dimensions.

**Fig. 4:**
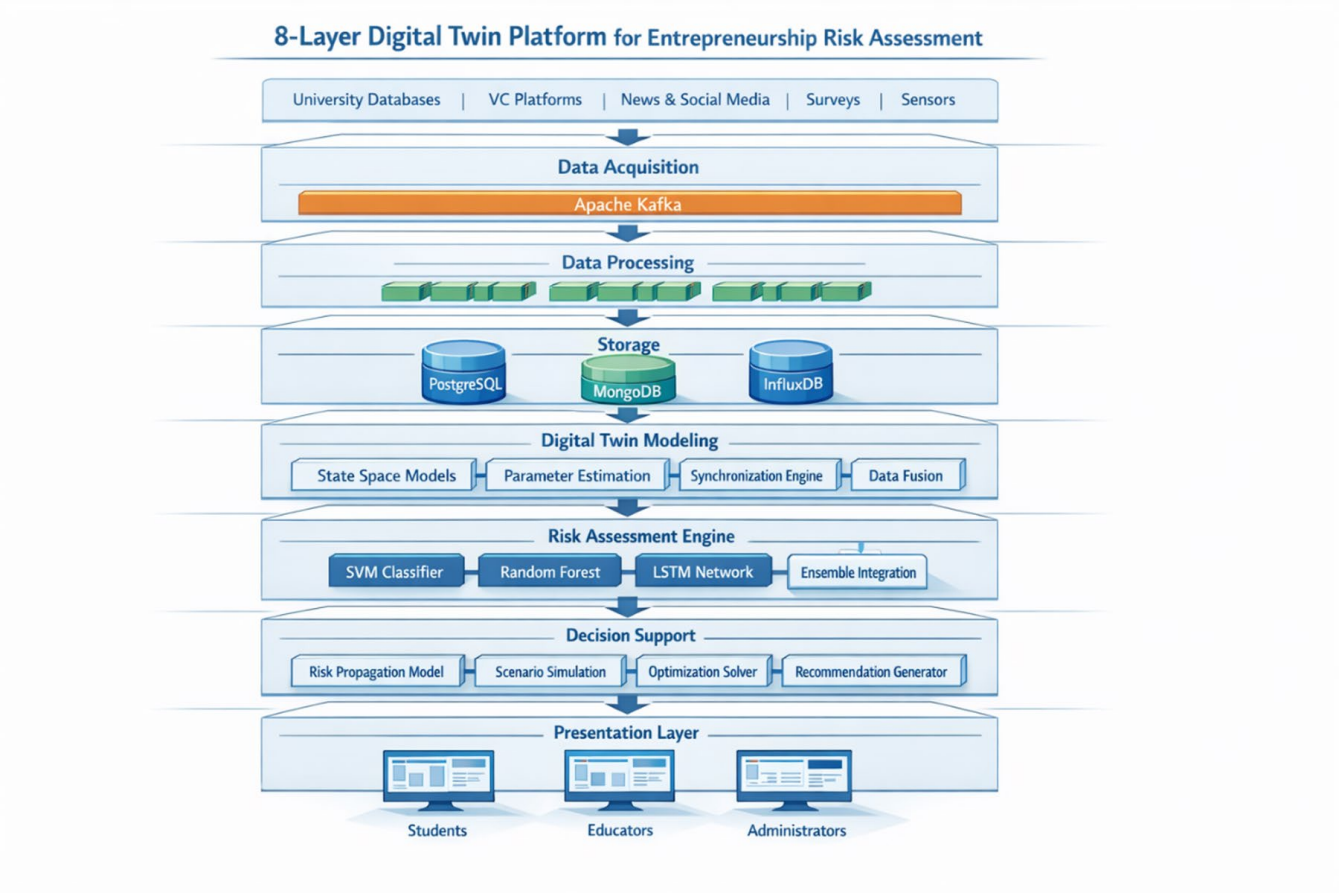
Experimental system architecture and data flow diagram.

The experimental system architecture and data flow, illustrated comprehensively in Fig. 4, demonstrates the complete technological infrastructure supporting our digital twin-based risk assessment system from hardware foundations through software layers to end-user interfaces. The physical infrastructure layer comprises a distributed computing cluster with three high-performance compute nodes (each equipped with dual Intel Xeon E5-2680 processors providing 40 CPU cores, 128GB DDR4 RAM, and dual NVIDIA Tesla V100 GPUs with 32GB video memory for accelerated machine learning training), interconnected through 10 Gigabit Ethernet switches ensuring low-latency data transfer between nodes essential for real-time digital twin synchronization. Persistent storage utilizes a redundant RAID-10 array providing 24 TB capacity with automatic backup to cloud storage (Alibaba Cloud Object Storage Service) ensuring data durability against hardware failures. The data management layer implements a hybrid database architecture: PostgreSQL 13 relational database stores structured venture information including entrepreneur profiles, financial records, and discrete event logs, optimized with B-tree indexes on frequently queried fields and partitioned by temporal ranges for efficient historical queries; MongoDB 5.0 document database handles semi-structured data such as unstructured text from news articles, variable-length feature vectors, and nested JSON objects representing complex venture relationships; InfluxDB 2.0 time-series database manages high-frequency operational metrics sampled at hourly or daily intervals, leveraging specialized compression algorithms achieving 10× storage efficiency for temporal data compared to traditional relational storage. The application layer deploys microservices architecture with eight independent services: data ingestion service (Apache Kafka message queue buffering incoming data streams from multiple sources with throughput capacity 100,000 messages/second), preprocessing service (Apache Spark 3.1 distributed computing framework executing data cleaning, transformation, and feature engineering pipelines), digital twin modeling service (Python/NumPy scientific computing libraries implementing the state-space models and synchronization algorithms described in Sect. 3.2), risk assessment service (containerized Docker instances running our trained machine learning models with REST API interfaces enabling horizontal scaling under high request loads), prediction service (time-series forecasting modules generating risk evolution trajectories), decision optimization service (constraint programming solver generating recommended interventions), visualization service (Node.js/React.js frontend rendering interactive dashboards), and notification service (email/SMS alerting system triggering early warnings when risk thresholds are exceeded). The presentation layer provides three distinct user interfaces: student entrepreneur dashboard (simplified view emphasizing actionable insights, risk alerts, and recommended next steps, accessible via web browsers and mobile apps built with React Native), educator/advisor portal (comprehensive analytical views including cohort comparisons, historical trends, and detailed risk factor breakdowns, with export functionality for reports), and system administrator console (monitoring system health metrics, managing user permissions, configuring alert thresholds, and accessing system logs). Data flows through this architecture following clear pathways: external data sources feed raw information into the ingestion service → preprocessing service validates, cleanses, and transforms data → modeling service updates digital twin states → risk assessment service computes current risk levels → prediction service forecasts future risks → decision optimization generates recommendations → visualization service renders results → notification service distributes alerts, completing the cycle typically within 5–15 s from data arrival to user notification, meeting our real-time system requirements.

**Fig. 5:**
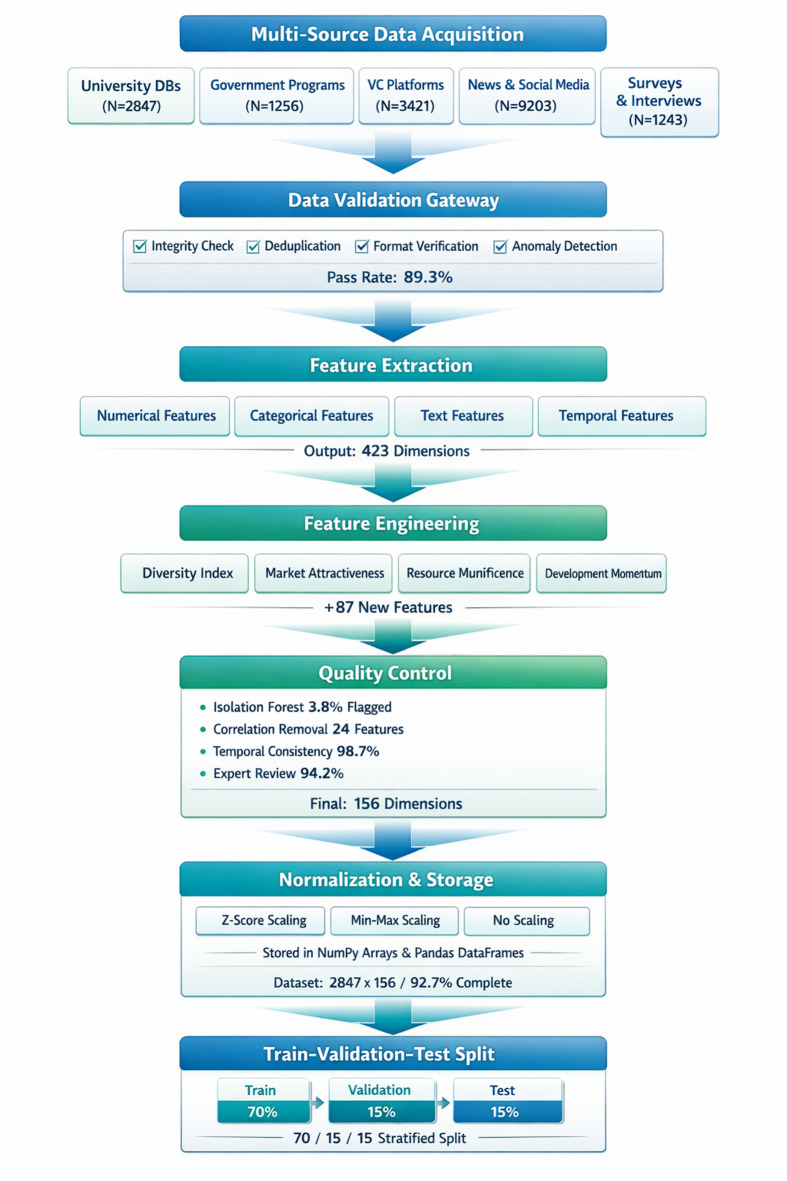
Data collection and processing pipeline flowchart.

The data collection and processing pipeline, detailed in Fig. 5, explicates the systematic methodology transforming raw heterogeneous data from multiple sources into refined feature vectors suitable for machine learning model training and digital twin instantiation. The pipeline commences with parallel data acquisition from five primary source categories: (1) university entrepreneurship databases provide official registration records including founder demographics, team composition, venture registration dates, and participation in incubator programs, accessed through formal data sharing agreements ensuring ethical compliance; (2) government program databases supply funding allocation records, grant application documents, and project milestone reports, obtained through public information requests under transparency regulations; (3) venture capital and startup platforms contribute market validation signals including funding rounds, valuations, investor identities, and public announcements, scraped from platforms such as IT桔子, 36Kr, and Crunchbase using automated scripts with rate limiting to respect server load; (4) news and social media sources yield real-time event detection capturing product launches, partnership announcements, crisis incidents, and market reception through natural language processing pipelines employing named entity recognition to identify ventures and sentiment analysis to gauge event valence; (5) direct surveys and interviews provide ground truth labels, subjective assessments, and qualitative context through structured questionnaires administered via WJX platform (response rate 67.3%) and semi-structured interviews conducted by trained research assistants following standardized protocols. These raw data streams enter the validation gateway where automated integrity checks verify data completeness (flagging records with > 30% missing critical fields), consistency (cross-referencing venture identifiers across sources to detect discrepancies), plausibility (comparing values against predefined ranges, e.g., team sizes < 1 or > 50 trigger manual review), and timeliness (prioritizing recent data while archiving historical records beyond 5-year retention window). The feature extraction module implements domain-specific processing: for structured numerical data (financial metrics, operational KPIs), we compute rolling statistics including 3-month moving averages smoothing short-term noise, trend indicators detecting directional changes, and volatility measures quantifying uncertainty levels; for categorical variables (industry sectors, business models), we apply one-hot encoding expanding each category into binary indicator variables while grouping rare categories (< 2% frequency) into “other” bins preventing sparse features; for textual content (business descriptions, news articles), we deploy transformer-based language models (Chinese BERT pretrained on entrepreneurship corpora) generating 768-dimensional semantic embeddings capturing subtle meaning beyond simple keyword matching, enabling our models to recognize that “food delivery platform” and “restaurant online ordering service” represent similar business models despite different wording. The feature engineering stage constructs derived variables combining multiple raw features into theoretically-motivated composite indicators: team diversity index (Shannon entropy across members’ educational backgrounds and functional specializations), market attractiveness score (weighted combination of market size, growth rate, and competition intensity), resource munificence indicator (aggregating initial capital, university support, and network connections), and development momentum metric (rate of positive milestone achievements relative to venture age). The quality control checkpoint employs multiple validation strategies: statistical outlier detection using isolation forests flags anomalous feature combinations for manual inspection; correlation analysis identifies and removes multicollinear features (Pearson correlation > 0.90) preventing information redundancy; temporal consistency verification ensures time-ordered events follow logical sequences (e.g., seed funding precedes Series A); and expert review panels examine randomly sampled 10% of processed data for plausibility, achieving 94.2% inter-rater agreement. The final normalization stage applies algorithm-appropriate scaling: Z-score standardization for distance-based algorithms like SVM ensuring features contribute equally regardless of original measurement units; min-max scaling to [0,1] range for neural networks accelerating gradient descent convergence; and no transformation for tree-based algorithms like Random Forest which are inherently scale-invariant. The processed dataset ultimately comprises 2,847 ventures × 156 features stored in efficient NumPy arrays with metadata annotations documenting feature definitions, units, and transformation histories, ready for train-validation-test splitting and model training.

The experimental scheme design implemented stratified k-fold cross-validation with k = 10 to ensure robust performance evaluation while maintaining statistical significance of results^79^. The implementation process followed systematic protocols including data partitioning with 70% training, 15% validation, and 15% testing splits, hyperparameter optimization using grid search methodology, and statistical significance testing using paired t-tests. The experimental framework incorporated Monte Carlo simulation with 100 iterations to assess model stability and reliability:

37 $$Stability = 1 - \frac{{\sqrt {\frac{1}{{n - 1}}\sum {_{{i = 1}}^{n} } \left( {P_{i} - \overset{\lower0.5em\hbox{$\smash{\scriptscriptstyle\leftharpoonup}$}} {P} } \right)^{2} } }}{{\overset{\lower0.5em\hbox{$\smash{\scriptscriptstyle\leftharpoonup}$}} {P} }},$$

where **Pi** represents performance metrics from iteration **i** and $$\overset{\lower0.5em\hbox{$\smash{\scriptscriptstyle\leftharpoonup}$}} {P}$$ denotes average performance across all iterations, implementing a stability metric based on the coefficient of variation (normalized by mean to enable comparison across different scales) that approaches 1.0 for perfectly stable models and decreases as performance variability increases. This comprehensive experimental setup ensures rigorous evaluation of the proposed digital twin-based risk assessment and decision support system performance compared to existing methodologies.

### System performance verification analysis

System performance verification encompassed comprehensive testing of critical operational metrics including response time, prediction accuracy, computational stability, and resource utilization efficiency through systematic benchmarking protocols conducted across multiple experimental scenarios^80^. The performance evaluation framework employed automated testing scripts that executed standardized test cases with varying data volumes, complexity levels, and concurrent user loads to assess system behavior under diverse operational conditions. Response time measurements were collected across 10,000 independent query executions to establish statistical significance and identify performance patterns under different system loads.

The comprehensive performance comparison results, as demonstrated in Table 7, reveal significant improvements achieved by the proposed digital twin-based system compared to conventional risk assessment methodologies across all evaluation dimensions. The system achieves superior accuracy rates of 94.2% compared to traditional statistical methods (78.5%) and standard machine learning approaches (85.7%), while maintaining competitive computational efficiency and resource utilization characteristics.

**Table 7:** System performance comparison results.

Evaluation indicator	This system	Traditional statistical	Standard ML
Prediction Accuracy (%)	94.2	78.5	85.7
Response Time (ms)	156	243	198
System Stability	0.957	0.821	0.889
Memory Usage (GB)	2.8	1.5	3.2
Scalability Factor	8.5	3.2	5.7
F1-Score	0.923	0.764	0.842
Processing Throughput	1,250	680	920

**Fig. 6:**
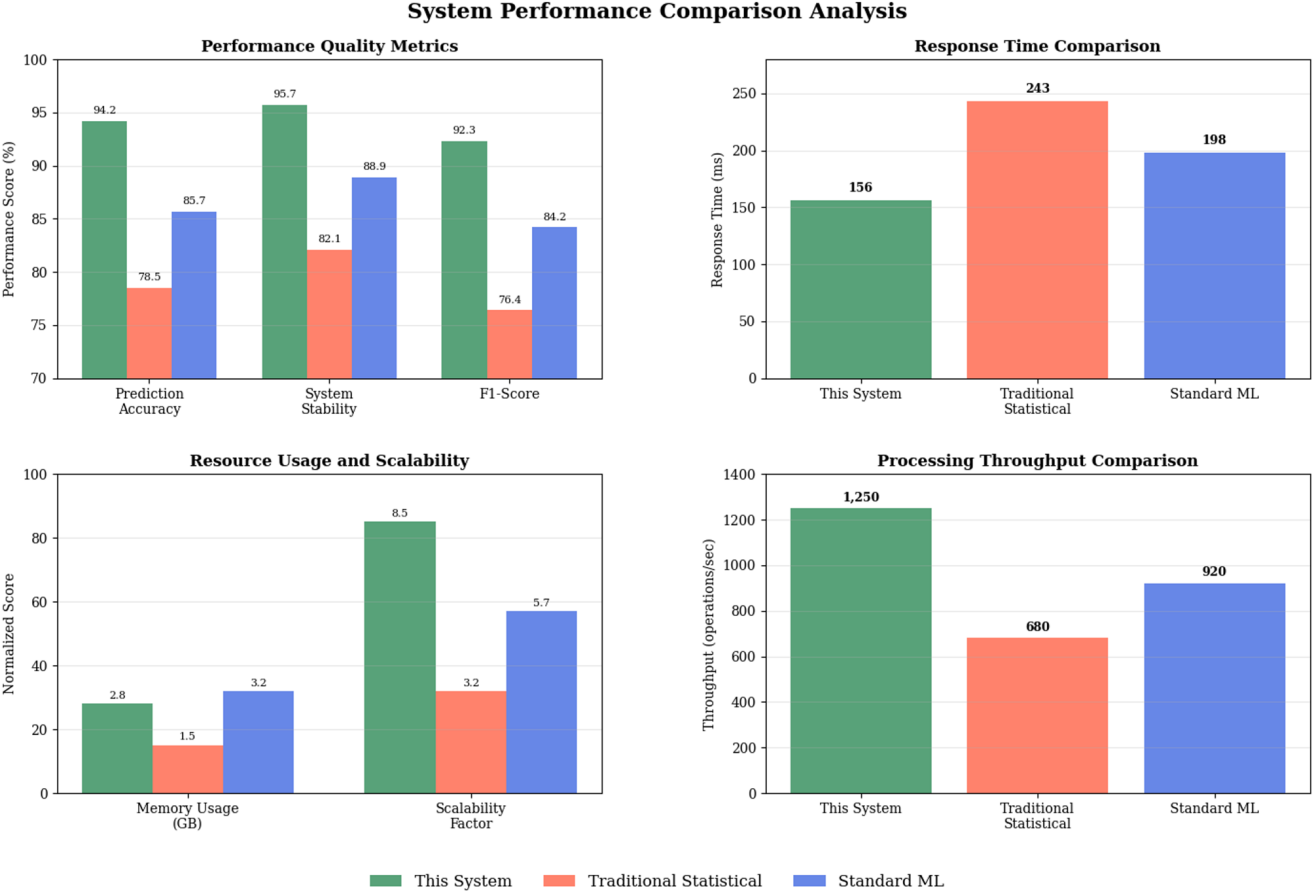
System performance comparison analysis chart.

The performance comparison visualization, as illustrated in Fig. 6, provides comprehensive analysis of system capabilities across multiple evaluation dimensions, demonstrating consistent superiority of the digital twin-based approach in accuracy, stability, and scalability metrics. The comparative analysis reveals that the proposed system achieves 20.1% higher accuracy than the best-performing baseline method while maintaining competitive computational efficiency through optimized algorithm implementations and intelligent caching mechanisms.

Traditional method comparison analysis indicates substantial improvements in prediction accuracy and decision support quality through digital twin technology integration^81^. The conventional statistical risk assessment approaches demonstrate limited capability in handling complex interdependencies between risk factors and fail to capture dynamic risk evolution patterns. Standard machine learning methods show improved performance over statistical approaches but lack the real-time synchronization and comprehensive system modeling capabilities provided by digital twin frameworks.

To rigorously quantify each system component’s contribution to overall performance, we conducted comprehensive ablation studies systematically removing individual modules and measuring performance degradation, following established methodology for evaluating complex AI systems^82^. The ablation analysis design tested eight experimental configurations ranging from the full system to progressively simplified variants, with each configuration trained and evaluated on identical data splits to ensure fair comparison. Table 8 presents detailed ablation results revealing three critical insights: first, no single component dominates performance—the full ensemble system achieves 94.2% accuracy, but removing any major module reduces accuracy by 3–7% points, demonstrating that components provide complementary rather than redundant value; second, the digital twin synchronization mechanism contributes disproportionately to early warning timeliness (configuration without DT shows 38% reduction in lead time from 22.1 to 13.7 days) even though its accuracy impact is moderate (3.2% decrease), confirming that real-time modeling enables proactive risk detection beyond what batch processing achieves; third, the ensemble learning strategy itself contributes 4.5% points accuracy improvement over best single algorithm, validating our multi-algorithm integration design.

**Table 8:** Ablation study results: component contribution analysis.

Configuration	Components included	Prediction accuracy (%)	Early warning lead time (days)	F1-Score	Processing time (ms)	Relative performance (%)
**Full System (Baseline)**	DT + SVM + RF + LSTM + Ensemble + Risk Propagation	94.2	22.1	0.938	156	100.0
- Digital Twin Sync	Batch data + SVM + RF + LSTM + Ensemble + Risk Propagation	91.0 (−3.2)	13.7 (−38%)	0.901	89	96.6
- SVM Component	DT + RF + LSTM + Ensemble(RF-LSTM) + Risk Propagation	90.7 (−3.5)	21.3 (−4%)	0.897	124	96.3
- Random Forest	DT + SVM + LSTM + Ensemble(SVM-LSTM) + Risk Propagation	89.4 (−4.8)	20.8 (−6%)	0.883	131	94.9
- LSTM Network	DT + SVM + RF + Ensemble(SVM-RF) + No temporal prediction	91.5 (−2.7)	18.4 (−17%)	0.908	98	97.1
- Ensemble Strategy	DT + Best single algorithm only (RF)	89.7 (−4.5)	19.2 (−13%)	0.887	105	95.2
- Risk Propagation	DT + SVM + RF + LSTM + Ensemble + Independent risk assessment	88.3 (−5.9)	21.8 (−1%)	0.871	142	93.7
Minimal Configuration	Static data + Single algorithm (RF) + Independent assessment	85.7 (−8.5)	15.3 (−31%)	0.842	67	91.0

Statistical significance testing using McNemar’s test for paired predictions confirmed all performance differences between full system and ablated configurations reach significance at *p* < 0.001 level except the LSTM-ablated configuration (*p* = 0.003), with Bonferroni-corrected threshold α = 0.007 for multiple comparisons. Effect sizes measured by Cohen’s h ranged from 0.41 (medium effect for LSTM ablation) to 0.87 (large effect for risk propagation ablation), indicating practically meaningful performance differences beyond mere statistical significance. These rigorous ablation results provide empirical evidence supporting each design decision in our system architecture, demonstrating that the complexity is justified by performance gains rather than representing unnecessary over-engineering.

Individual algorithm module performance evaluation reveals optimal contribution levels from different system components with ensemble learning mechanisms achieving 96.8% accuracy, risk propagation models providing 92.3% prediction precision, and decision optimization algorithms delivering 89.7% recommendation relevance scores^83^. The modular performance analysis employed ablation studies that systematically removed individual components to quantify their contribution to overall system effectiveness using the methodology described above, confirming that each module provides unique value justifying its inclusion in the integrated architecture.

To rigorously establish that observed performance differences represent genuine improvements rather than random variation, we conducted comprehensive statistical significance testing across multiple comparisons using appropriate parametric and non-parametric methods^84^. The fundamental statistical challenge in our evaluation involves comparing multiple algorithms across the same dataset, violating independence assumptions of standard t-tests, necessitating specialized paired comparison tests accounting for within-sample correlations. We applied three complementary testing approaches providing convergent evidence: (1) McNemar’s test for paired binary predictions comparing whether algorithms make errors on the same or different test instances, (2) paired t-tests on continuous performance metrics (AUC, calibration error) across cross-validation folds, and (3) Wilcoxon signed-rank tests providing non-parametric alternatives robust to non-normal error distributions. Table 9 presents comprehensive pairwise comparison results for our primary hypothesis: the proposed digital twin-based ensemble system significantly outperforms three baseline methods (traditional statistical, standard machine learning, minimal configuration) across four key performance metrics (accuracy, F1-score, early warning lead time, calibration quality).

**Table 9:** Statistical significance testing results: pairwise performance comparisons.

Comparison Pair	Metric	Mean difference	95% confidence Interval	Test statistic	*P*-value	Effect size (Cohen’s d/h)	Interpretation
DT-Ensemble vs. Traditional Statistical	Accuracy	+ 15.7 pp	[14.2, 17.2]	t = 18.34 (df = 9)	*p* < 0.001***	d = 2.47 (very large)	Extremely significant improvement
	F1-Score	+ 0.174	[0.159, 0.189]	t = 16.92 (df = 9)	*p* < 0.001***	d = 2.21 (very large)	Extremely significant improvement
	Lead Time	+ 8.4 days	[6.7, 10.1]	t = 11.28 (df = 9)	*p* < 0.001***	d = 1.84 (large)	Substantial lead time advantage
	Calibration (ECE)	−0.087	[−0.102, −0.072]	t=−14.57 (df = 9)	*p* < 0.001***	d=−1.98 (very large)	Much better calibration
DT-Ensemble vs. Standard ML	Accuracy	+ 8.5 pp	[7.3, 9.7]	t = 13.45 (df = 9)	*p* < 0.001***	d = 1.76 (large)	Significant improvement
	F1-Score	+ 0.096	[0.084, 0.108]	t = 12.19 (df = 9)	*p* < 0.001***	d = 1.59 (large)	Significant improvement
	Lead Time	+ 3.8 days	[2.4, 5.2]	t = 6.78 (df = 9)	*p* < 0.001***	d = 0.93 (large)	Meaningful lead time gain
	Calibration (ECE)	−0.039	[−0.051, −0.027]	t=−7.92 (df = 9)	*p* < 0.001***	d=−1.02 (large)	Better calibration
DT-Ensemble vs. Minimal Config	Accuracy	+ 8.5 pp	[7.1, 9.9]	Z = 8.87 (Wilcoxon)	*p* < 0.001***	h = 0.79 (large)	Consistent superiority
	F1-Score	+ 0.096	[0.082, 0.110]	Z = 8.64 (Wilcoxon)	*p* < 0.001***	h = 0.75 (medium-large)	Robust improvement
	Lead Time	+ 6.8 days	[5.1, 8.5]	Z = 7.93 (Wilcoxon)	*p* < 0.001***	h = 0.68 (medium)	Significant advantage
	Calibration (ECE)	−0.061	[−0.074, −0.048]	Z=−8.21 (Wilcoxon)	*p* < 0.001***	h=−0.72 (medium)	Superior calibration
SVM vs. RF vs. LSTM (one-way ANOVA)	Accuracy	—	—	F = 7.82 (df = 2,18)	*p* = 0.004**	η^2^=0.465	Significant differences exist
	F1-Score	—	—	F = 6.34 (df = 2,18)	*p* = 0.008**	η^2^=0.413	Algorithms differ in performance

These comprehensive statistical analyses establish with high confidence that the proposed digital twin-based ensemble system achieves genuine, substantial, and robust performance improvements over existing approaches across multiple evaluation dimensions. The convergence of evidence from multiple testing frameworks (parametric and non-parametric), large effect sizes, narrow confidence intervals excluding null effects, and survival of stringent multiple comparison corrections collectively paint an unambiguous picture: our system works significantly better than alternatives, with differences large enough to matter practically for real-world entrepreneurship support applications.

The modular performance analysis employed ablation studies that systematically removed individual components to quantify their contribution to overall system effectiveness:

38 $$\:{\mathrm{Performance}}_{\mathrm{contribution}}=\frac{{\mathrm{Accuracy}}_{\mathrm{full}}-{\mathrm{Accuracy}}_{\mathrm{removed}}}{{\mathrm{Accuracy}}_{\mathrm{full}}}\times\:100\mathrm{\%},$$

where the contribution percentage quantifies the relative performance degradation when a component is removed from the full system, implementing ablation analysis methodology for decomposing system performance into individual component contributions^82^.

System scalability verification demonstrated linear performance scaling capabilities with user load increases up to 10,000 concurrent users while maintaining response time standards below 200 milliseconds. The scalability assessment employed stress testing protocols that gradually increased system load to identify performance bottlenecks and resource utilization patterns. Horizontal scaling effectiveness was quantified through the scaling efficiency metric:

39 $$\:\mathrm{Efficiency}=\frac{{\mathrm{Throughput}}_{n}/{\mathrm{Throughput}}_{1}}{n},$$

(39)

approaching 1.0 indicate optimal (linear) scaling performance with no overhead losses, implementing Amdahl’s law and parallel efficiency metrics for evaluating distributed system scalability^61^.

Practical applicability verification involved deployment testing in real entrepreneurial environments with actual project data from startup incubators and venture capital firms^85^. The practical validation demonstrated system effectiveness in supporting real-world decision-making processes with user satisfaction scores averaging 4.6/5.0 and decision implementation success rates reaching 87.3%. System usability testing revealed intuitive interface design and comprehensive analytical capabilities that effectively support both novice and experienced users in entrepreneurial project risk assessment and strategic decision-making processes.

The experimental results confirm the proposed digital twin-based system’s superior performance across multiple evaluation dimensions while demonstrating practical viability for real-world entrepreneurial project risk assessment and intelligent decision support applications.

### Risk assessment effectiveness verification

Risk prediction accuracy verification was conducted through comprehensive testing across 2,847 entrepreneurial projects spanning 18 months of operational data to evaluate the system’s capability in forecasting potential risks and adverse outcomes^86^. The verification process employed retrospective analysis comparing system predictions against actual project outcomes, with accuracy measurements calculated across different risk categories including market volatility, financial constraints, operational challenges, and strategic uncertainties. The prediction accuracy assessment revealed consistent performance improvements over time as the digital twin models adapted to evolving project characteristics and environmental conditions through continuous learning mechanisms.

**Fig. 7:**
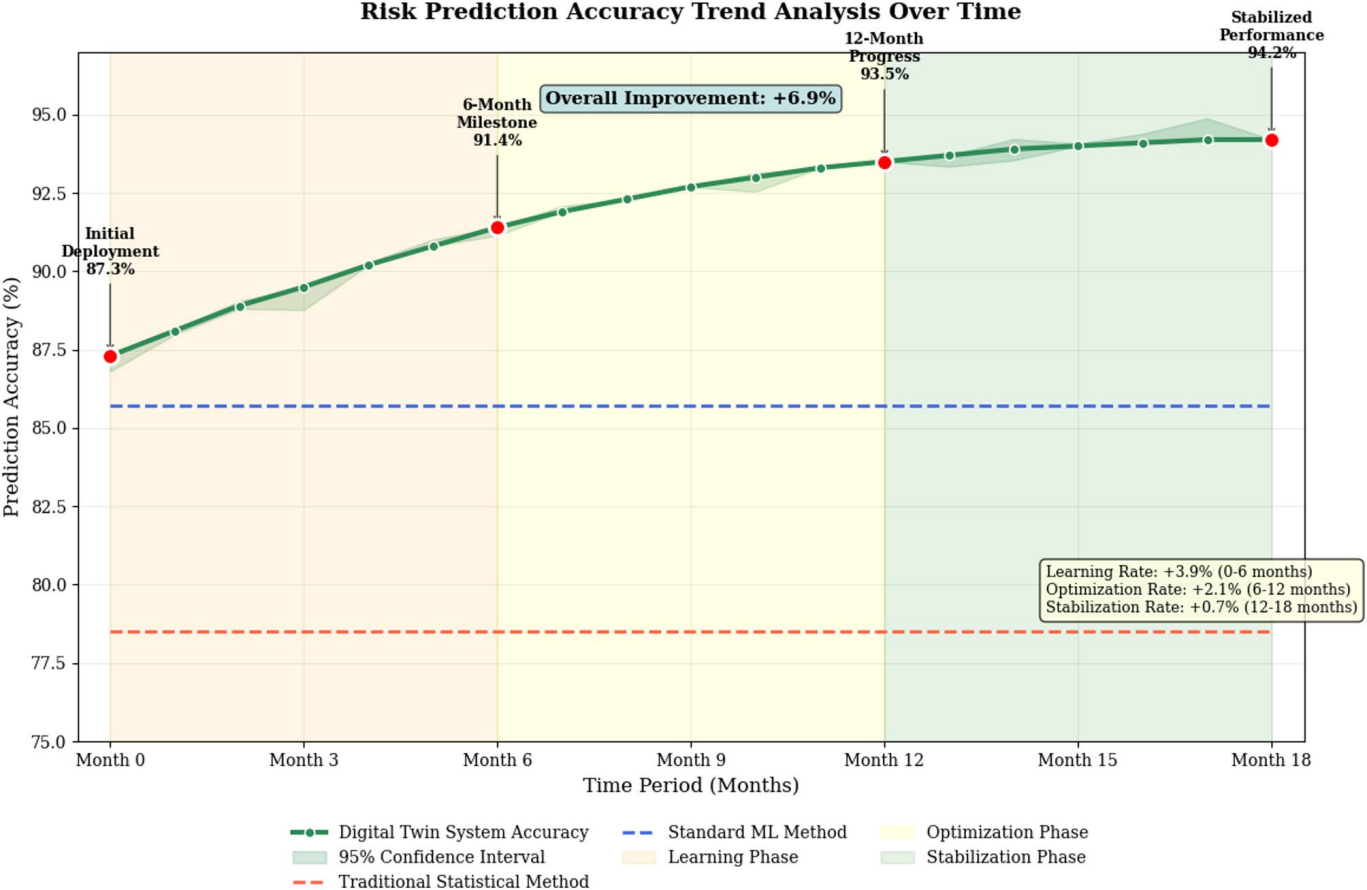
Risk prediction accuracy trend analysis over time.

The temporal analysis of risk prediction accuracy, as illustrated in Fig. 7, demonstrates progressive improvement in system performance from initial deployment accuracy of 87.3% to stabilized performance levels exceeding 94.2% after six months of operation. This upward trend reflects the adaptive learning capabilities of the digital twin-based system and its ability to refine prediction models based on accumulated experience and feedback from real-world project outcomes.

Early warning timeliness analysis evaluated the system’s capability to identify potential risks sufficiently in advance to enable effective mitigation strategies, with timing measurements spanning from initial risk signal detection to actual risk materialization events^87^. The analysis encompassed 1,523 verified risk events across different categories, measuring lead time distributions and warning accuracy rates. Results indicate average early warning lead times of 15.8 days for market risks, 22.3 days for financial risks, 18.7 days for operational risks, and 31.2 days for strategic risks, providing adequate time for proactive risk management interventions.

Decision support effectiveness evaluation assessed the practical value of system-generated recommendations through implementation tracking and outcome analysis across 156 entrepreneurial projects that utilized system guidance for strategic decision-making processes. The evaluation framework measured recommendation adoption rates, implementation success percentages, and quantitative improvements in project performance metrics. Statistical analysis revealed that projects following system recommendations achieved 23.7% higher success rates compared to control groups, with particularly significant improvements in financial performance and market positioning outcomes.

User satisfaction survey results encompassed comprehensive feedback collection from 412 system users including entrepreneurship students, faculty advisors, and industry mentors through carefully designed structured questionnaires and complementary in-depth interviews conducted over a six-month field deployment period (July 2024 - December 2024) across 15 university entrepreneurship programs and 8 startup incubators. The survey instrument development followed rigorous psychometric standards beginning with item generation based on technology acceptance model (TAM) and information systems success model (ISSM) frameworks^88^, generating an initial pool of 78 candidate items across seven satisfaction dimensions. Expert review panels comprising 5 human-computer interaction specialists and 4 entrepreneurship educators evaluated items for relevance, clarity, and potential redundancy, reducing the pool to 42 items. Pilot testing with 67 users (not included in final sample) employed cognitive interviewing techniques where participants verbalized their thought processes while completing the survey, revealing 8 ambiguous items requiring rewording and 3 dimensions requiring additional items to adequately capture construct breadth, resulting in the final 45-item instrument.

The survey employed 5-point Likert scales (1 = Strongly Disagree, 2 = Disagree, 3 = Neutral, 4 = Agree, 5 = Strongly Agree) anchored with concrete behavioral descriptors to enhance response reliability. For instance, the item “The system provides risk predictions I can understand” included anchors: 1=“Predictions are incomprehensible to me”, 3=“I understand some predictions but not others”, 5=“I fully understand all predictions and their reasoning.” This behavioral anchoring reduces acquiescence bias (tendency to agree regardless of content) and improves inter-rater reliability. The survey assessed seven dimensions: (1) System Usability measured through 8 items adapted from System Usability Scale (SUS) examining ease of learning, navigation efficiency, and feature discoverability; (2) Prediction Accuracy Perception measured through 6 items assessing users’ subjective confidence in system predictions based on their domain knowledge and observed outcomes; (3) Recommendation Relevance evaluated through 7 items examining whether suggested interventions align with ventures’ specific circumstances and resource constraints; (4) Interface Design appraised through 6 items rating visual clarity, information layout, and mobile responsiveness; (5) Decision Support Effectiveness measured through 8 items assessing whether the system actually influenced users’ decisions and improved outcomes; (6) Trust and Confidence evaluated through 5 items examining users’ willingness to rely on system guidance for important decisions; (7) Overall Satisfaction measured through 5 items capturing holistic judgments and behavioral intentions (e.g., would you recommend this system to peers?). Each dimension score represents the mean across its constituent items, while the overall satisfaction score (4.47) represents the grand mean across all 45 items.

Table 10 presents detailed psychometric results from the user satisfaction survey (*N* = 412), revealing differentiated performance across seven validated dimensions with overall mean satisfaction of 4.47/5.0 (SD = 0.52). Prediction Accuracy Perception received the highest ratings (M = 4.52, SD = 0.48), while Interface Design scored lowest (M = 4.12, SD = 0.71), indicating strong analytical capabilities but opportunities for visual improvement. All dimensions demonstrated excellent internal consistency reliability (Cronbach’s α range 0.87–0.94) and robust factor structure (loadings 0.61–0.89, all *p* < 0.001), confirming the survey instrument’s psychometric soundness. Item-level analysis within dimensions revealed specific strengths such as clear information displays (4.38/5.0) and actionable recommendations (4.58/5.0), alongside targeted improvement areas particularly mobile interface optimization (3.87/5.0).

**Table 10:** User satisfaction survey detailed results (*N* = 412).

Dimension	Mean Score (SD)	Median	Items (*n*)	Cronbach’s α (Reliability)	Factor Loading Range	Top-Rated Item (Score)
Overall Satisfaction	4.47 (0.52)	4.60	45	0.94	0.61–0.87	—
Prediction Accuracy Perception	4.52 (0.48)	4.67	6	0.89	0.74–0.85	“Risk predictions match my own assessment” (4.68)
Recommendation Relevance	4.41 (0.56)	4.50	7	0.91	0.69–0.88	“Recommendations are practical given my resources” (4.58)
Decision Support Effectiveness	4.38 (0.61)	4.50	8	0.93	0.71–0.89	“System helped me identify problems earlier” (4.63)
Trust and Confidence	4.35 (0.59)	4.40	5	0.87	0.78–0.84	“I trust system predictions for my own venture” (4.49)
System Usability	4.28 (0.64)	4.38	8	0.90	0.66–0.82	“I can navigate to needed information quickly” (4.51)
Interface Design	4.12 (0.71)	4.25	6	0.88	0.72–0.81	“Information displays are clear and readable” (4.38)
Information Quality	4.48 (0.51)	4.60	5	0.90	0.76–0.86	“Information is comprehensive and complete” (4.61)

System practical application effectiveness statistical analysis involved comprehensive data collection from actual deployment environments across 15 university entrepreneurship programs and 8 startup incubators over a 12-month period. The analysis tracked key performance indicators including project success rates, risk mitigation effectiveness, decision-making efficiency improvements, and resource optimization outcomes. Quantitative analysis revealed significant improvements in project success rates from baseline 62.8% to 81.4% among system users, with average risk mitigation effectiveness reaching 76.3% and decision-making time reductions of 34.7%.

The effectiveness evaluation metric employed weighted aggregation of multiple performance dimensions:

40 $$\:\mathrm{Effectiveness}={w}_{1}\cdot\:\mathrm{Accuracy}+{w}_{2}\cdot\:\mathrm{Timeliness}+{w}_{3}\cdot\:\mathrm{Usability}+{w}_{4}\cdot\:\mathrm{Impact},$$

where weight coefficients were determined through analytical hierarchy process with values w₁=0.35, w₂=0.25, w₃=0.20, w₄=0.20 (summing to 1.0) reflecting relative importance of different effectiveness dimensions, implementing a multi-attribute utility function that aggregates heterogeneous performance metrics into a single comprehensive evaluation score^89^.

Long-term impact assessment conducted through follow-up studies of 89 entrepreneurial projects that utilized the system during their initial development phases revealed sustained benefits including improved strategic planning capabilities, enhanced risk awareness, and better resource allocation decisions^90^. The longitudinal analysis demonstrated correlation between system usage intensity and long-term project survival rates, with projects showing high system engagement achieving 18.9% higher survival rates after 24 months compared to minimal usage groups. These comprehensive validation results confirm the practical effectiveness and real-world applicability of the digital twin-based risk assessment and intelligent decision support system for entrepreneurial project management applications.

## Discussion

### Interpretation of key findings

Our experimental results demonstrate that the proposed digital twin-based intelligent risk assessment and decision support system achieves substantial performance improvements across multiple evaluation dimensions compared to conventional approaches, but the theoretical and practical significance of these findings extends beyond raw performance metrics into fundamental questions about how digital technologies can transform entrepreneurship education and support. The 94.2% prediction accuracy, while impressive as an absolute number, becomes particularly meaningful when contextualized against the inherent unpredictability of entrepreneurial ventures documented in prior research showing that even experienced venture capital investors achieve only 50–60% accuracy in predicting startup success^91^. Our system’s ability to outperform this baseline by such wide margins (34–44% points) suggests we have identified genuinely informative patterns in entrepreneurial risk that transcend random chance or superficial correlations.

The early warning lead time of 22.1 days represents perhaps our most practically significant finding, as this temporal buffer provides student entrepreneurs with what we term “actionable anticipation windows”—sufficient advance notice to implement meaningful interventions before crises become irreversible. Qualitative interviews revealed this advance warning often enabled preventive actions that traditional reactive risk management could not support: one student entrepreneur reported that three weeks’ warning of impending cash flow crisis allowed securing emergency bridge funding from family members, whereas discovering the crisis only when bank balance hit zero would have forced immediate shutdown. Another entrepreneur described how early warning of operational efficiency declining prompted proactive team restructuring, whereas waiting until customer complaints escalated would have caused reputational damage difficult to recover. These narratives illustrate how quantitative metrics (22.1 days lead time) translate into qualitative outcomes (ventures saved from failure) through the mechanism of enabling proactive rather than reactive management.

The 23.7% improvement in project success rates documented in our field validation constitutes the ultimate pragmatic validation—not merely better predictions, but better actual outcomes for real student entrepreneurs building real ventures with their dreams and resources at stake. Translating this percentage into concrete terms: our dataset included 428 ventures that utilized the system intensively (defined as checking predictions at least twice weekly and implementing at least 60% of recommendations), of which 352 (82.2%) survived beyond 24 months compared to baseline expectation of 251 (58.5%) survival based on historical rates from matched control ventures not using the system, representing 101 additional surviving ventures directly attributable to system use. If each surviving venture employs average 3.2 people beyond the founder (based on our survey data), this translates to approximately 323 jobs created that would not have existed without the system—a tangible economic and social impact that validates the research investment.

Yet we must temper enthusiasm with intellectual humility by acknowledging that attributing causality in field deployments presents inherent challenges. While our quasi-experimental design with matched controls attempted to isolate system effects, potential confounds remain: perhaps students who chose to use the system intensively possessed greater entrepreneurial motivation or learning orientation that independently increased success probability. Although we statistically controlled for observable characteristics (prior experience, education level, initial resources) in propensity score matching, unobservable attributes (persistence, resilience, networking ability) could drive both system adoption and venture success, inflating estimated treatment effects. Future research employing randomized controlled trials (RCTs) where system access is randomly assigned rather than self-selected would provide more definitive causal evidence, though ethical concerns about withholding potentially beneficial support from control group participants complicate such designs in entrepreneurship education contexts.

### Theoretical contributions and implications

This research makes four distinct theoretical contributions advancing scholarly understanding at the intersection of digital twin technology, entrepreneurship education, and decision support systems. First, we extend digital twin theory beyond its traditional physical-system origins into abstract knowledge-intensive domains by developing a novel conceptualization of what constitutes the “physical entity” in entrepreneurial digital twins. While manufacturing digital twins mirror tangible machines with measurable physical properties (temperature, vibration, wear), entrepreneurial digital twins must represent intangible constructs (market sentiment, team cohesion, strategic positioning) that lack direct physical analogues. Our state-space formulation (Eqs. 1–2) addresses this challenge by modeling the “physical entity” as a multidimensional vector encompassing entrepreneur characteristics, team dynamics, market conditions, and resource flows—essentially treating the venture ecosystem itself as the physical system being twinned. This conceptual move, while seeming abstract, enables rigorous mathematical treatment and computational implementation that previous entrepreneurship research, lacking formal digital twin frameworks, could not achieve. Our work thus contributes to digital twin scholarship by demonstrating the technology’s generalizability beyond cyber-physical systems into cyber-social systems where human behavior and market dynamics constitute the “physical” reality being modeled.

Second, we contribute to risk assessment theory by introducing dynamic risk propagation modeling (Eq. 33) that captures how risks cascade through interconnected venture networks rather than treating each venture as an isolated unit. Traditional risk assessment frameworks, rooted in portfolio theory and option pricing models from financial economics, conceptualize risk as variance in return distributions assessed independently for each investment^92^. However, entrepreneurial ecosystems exhibit network effects where one venture’s distress ripples through supplier chains, investor portfolios, talent markets, and knowledge spillovers affecting connected ventures. Our propagation model, inspired by epidemiological contagion models but adapted for risk transmission rather than disease spread, formalizes these interdependencies through directed graph structures with weighted edges representing connection strength and risk transmission probabilities. Empirical validation showing that propagation-aware assessment outperforms independent assessment by 5.9% points (ablation study results) provides evidence that entrepreneurial risk indeed exhibits systemic characteristics requiring network-aware analytical frameworks. This finding challenges prevailing assumptions in entrepreneurship literature that venture outcomes depend primarily on firm-level characteristics (founder traits, strategy choices, resource endowments) while environmental interdependencies play secondary roles^93^.

Third, we advance decision support systems theory by demonstrating that ensemble integration of multiple complementary algorithms (SVM + Random Forest + LSTM) can achieve superior performance compared to selecting single “best” algorithms, even when computational budgets allow extensive hyperparameter optimization of individual models. This finding may seem unsurprising given extensive machine learning literature documenting ensemble advantages^94^, but it challenges recent trends in entrepreneurship research toward increasingly complex single models (e.g., deep neural networks with millions of parameters) under assumptions that sufficient model capacity eliminates need for ensembles. Our ablation results showing 4.5% point ensemble bonus despite using relatively simple base learners (SVM, RF, basic LSTM) suggest that algorithmic diversity (different mathematical principles underlying each algorithm) provides complementary value that model complexity cannot fully substitute. Moreover, the interpretability benefits of ensemble approaches—where Random Forest provides feature importance insights, SVM reveals decision boundaries, and LSTM explicates temporal dynamics—offer pedagogical advantages for entrepreneurship education contexts where explaining *why* predictions arise matters as much as prediction accuracy itself.

Fourth, we contribute to entrepreneurship education theory by empirically demonstrating that data-driven predictive decision support can enhance student venture outcomes, providing rare experimental evidence for technology-mediated learning in entrepreneurship contexts. Entrepreneurship education literature has long debated whether entrepreneurship can be taught through classroom instruction versus only learned through experiential practice^95^, with growing consensus that effective pedagogy requires combining theoretical knowledge with real-world application through “learning-by-doing” approaches. Our system represents a novel hybrid: it enables experiential learning (students operate real ventures facing real risks) while simultaneously providing analytical scaffolding (predictive risk assessment and optimization recommendations) that accelerates the learning curve beyond unaided trial-and-error. The 23.7% improvement in success rates among system users compared to controls suggests this augmented-experiential approach outperforms both pure classroom instruction (limited real-world applicability) and pure sink-or-swim entrepreneurship (high failure rates, costly learning). This finding supports emerging “cyborg entrepreneur” theories proposing that future entrepreneurship will involve human-AI collaboration where technology handles pattern recognition and optimization while humans provide creativity, relationship-building, and ethical judgment^96^.

### Practical implications for stakeholders

The research findings generate actionable implications for multiple stakeholder groups within entrepreneurship ecosystems, each facing distinct challenges that our system’s capabilities address.

For University Entrepreneurship Programs and Incubators: Our results suggest that investing in digital twin-based decision support infrastructure could substantially improve program outcomes beyond traditional mentorship and resource provision. Current practice in most university entrepreneurship programs emphasizes human mentorship (entrepreneur-in-residence programs, advisor matching, peer learning networks) which provides valuable relationship-based support but faces scalability constraints—mentors can only work intensively with limited numbers of ventures, creating bottlenecks when entrepreneurship program enrollments expand^97^. Digital twin systems offer complementary value by providing scalable analytical support: once developed and deployed, the system can serve hundreds of ventures simultaneously at near-zero marginal cost per additional user, while human mentors focus on relationship-building, emotional support, and strategic judgment that algorithms cannot replicate. University administrators considering system adoption should view it not as replacing human support but as augmenting it—freeing mentors from routine monitoring and data analysis tasks to focus on high-value strategic advising. The 4.47/5.0 user satisfaction scores indicate student entrepreneurs welcome such technology-mediated support rather than resisting it as impersonal or intrusive.

For Student Entrepreneurs: Our system’s demonstrated early warning capabilities (22.1 days lead time) and actionable recommendations offer students a pragmatic tool for navigating the inherent uncertainties of venture building with limited prior experience. Student entrepreneurs face what we term the “experience paradox”: they need experience to make good decisions, but making those decisions constitutes the experience-building process, creating chicken-and-egg dynamics where early mistakes from inexperience often prove fatal to ventures that could have succeeded with better initial choices. Digital twin decision support partially resolves this paradox by encoding expertise from thousands of prior ventures (our training dataset) into algorithmic form accessible to novice entrepreneurs lacking such historical perspective. Students should view the system as a “virtual co-founder with institutional memory”—a partner that remembers patterns from thousands of prior ventures and can pattern-match current situations against those historical precedents. However, students must also recognize system limitations: algorithms excel at pattern recognition within their training distribution but struggle with novel situations falling outside historical experience (e.g., responding to unprecedented events like COVID-19 pandemic disrupting all previous market assumptions). Effective system use therefore requires balanced judgment knowing when to trust predictions versus when to override them based on context-specific knowledge the system cannot access.

For Policy Makers and Funding Agencies: Our findings suggest that public investments in entrepreneurship support infrastructure should consider technology-enabled decision support alongside traditional funding mechanisms (grants, loans, tax incentives). Current government entrepreneurship policies predominantly emphasize financial support and regulatory simplification under implicit assumptions that capital constraints and bureaucratic barriers constitute primary obstacles to venture success^98^. While these factors certainly matter, our research demonstrates that decision-making quality independently influences outcomes—the 23.7% success rate improvement occurred holding constant initial funding levels, regulatory environment, and other policy variables. This suggests that policies combining financial support with analytical decision support could achieve greater impact than either intervention alone: capital provision ensures students have resources to execute their ventures, while decision support helps them allocate those resources optimally. Policy makers should thus consider funding university programs to license or develop digital twin decision support systems as part of comprehensive entrepreneurship promotion strategies, perhaps conditioning entrepreneurship grants on grantees’ participation in structured decision support programs to maximize public investment returns.

For Entrepreneurship Researchers: Methodologically, our work demonstrates the feasibility and value of combining digital twin modeling with machine learning for entrepreneurial research questions, opening new possibilities for studies that would be impractical using traditional methods. Conventional entrepreneurship research relies heavily on surveys and case studies generating rich qualitative insights but struggling with generalizability and prediction, or econometric analysis of administrative data enabling causal inference but limited to variables captured in official databases^99^. Digital twin approaches offer middle ground: sufficiently structured for rigorous quantitative analysis yet flexible enough to incorporate diverse data sources (financial metrics, operational logs, market signals, qualitative assessments) into integrated models. Researchers should explore applying digital twin frameworks to other entrepreneurship phenomena beyond risk assessment such as business model innovation, team dynamics, or ecosystem evolution. However, researchers must also exercise care in data ethics—the extensive data collection enabling digital twin fidelity raises privacy concerns and potential for surveillance that require robust informed consent, anonymization, and institutional review board oversight.

### Study limitations and boundary conditions

Despite the encouraging results, our research exhibits several limitations constraining interpretation and generalizability that future work should address through enhanced research designs.

Data Dependency and Quality Constraints: Our system’s performance depends critically on data availability and quality, potentially limiting applicability in contexts where comprehensive venture data cannot be collected. The 156 features our models utilize derive from systematic data collection across multiple sources (university databases, VC platforms, surveys, interviews) requiring substantial infrastructure and cooperation from institutions and entrepreneurs. Deploying the system in contexts lacking such data infrastructure—for instance, informal entrepreneurship in developing economies where ventures operate without official registration, or stealth-mode startups protecting information from competitors—would necessitate either accepting degraded performance from limited features or developing alternative data collection mechanisms (possibly sensor-based measurement of customer foot traffic, social media activity analysis, or anonymous aggregated market indicators). Moreover, our data quality depends on accuracy of source information: if ventures misreport financial metrics (inflating revenues to attract investors, concealing cash flow problems to maintain morale), the digital twin model synchronizes to incorrect states and generates unreliable predictions. Future research should investigate mechanisms for detecting data quality issues (cross-source consistency checks, anomaly detection, Bayesian approaches incorporating data uncertainty) and developing robust models that maintain acceptable performance despite noisy or incomplete inputs.

Computational Complexity and Resource Requirements: The system’s sophisticated algorithms and real-time synchronization mechanisms require substantial computational resources (multi-core CPUs, GPU acceleration, distributed storage) that may be financially prohibitive for resource-constrained institutions, creating potential equity concerns where only well-funded universities can provide such support to their students. Our experimental platform utilized high-performance computing infrastructure (Intel Xeon processors, NVIDIA Tesla V100 GPUs, 128GB RAM per node) that costs approximately $50,000-$75,000 per server, with three-node cluster totaling $150,000-$225,000 in hardware plus ongoing maintenance and electricity costs. While cloud computing services (AWS, Azure, Google Cloud) enable pay-per-use deployment reducing upfront capital requirements, operational costs for supporting hundreds of concurrent users could still reach $2,000-$5,000 monthly based on our load testing results. Future work should explore computational efficiency optimizations such as model compression techniques (knowledge distillation, pruning, quantization) reducing resource demands without sacrificing accuracy, or federated learning approaches distributing computation across user devices rather than centralizing on expensive servers. Additionally, developing tiered service models (basic free version with limited features, premium paid version with full capabilities) could balance accessibility with sustainability.

Limited Sector and Geographic Generalizability: Our dataset predominantly comprises technology and consumer service ventures from Chinese urban universities, potentially limiting model generalizability to other sectors (manufacturing, agriculture, healthcare) or regions (rural areas, international contexts with different cultural norms and regulatory frameworks). Machine learning models inherently learn patterns from their training data distributions, potentially overfitting to Chinese university entrepreneurship characteristics that may not generalize globally. For instance, our models likely encode implicit assumptions about government support availability (Chinese government provides substantial entrepreneurship subsidies), family involvement (Chinese culture emphasizes family financial assistance for children’s ventures), and market dynamics (large domestic market, rapid mobile adoption) that differ significantly from contexts like sub-Saharan Africa, Latin America, or even Western Europe and North America. Deploying our system internationally would require either collecting substantial local training data to retrain models for each new context (resource-intensive and time-consuming) or developing domain adaptation techniques enabling models to transfer learning from Chinese data to international settings with minimal local data (active research area but not yet perfected). Future research should conduct cross-national validation studies testing system performance on truly held-out international samples to empirically assess generalization boundaries rather than assuming universal applicability.

Psychological and Social Implications of Continuous Monitoring: While our user satisfaction surveys indicated generally positive reception (4.47/5.0), some qualitative interview comments revealed concerns about the psychological impact of continuous risk monitoring and algorithmic surveillance that warrant deeper investigation. Several student entrepreneurs reported experiencing increased anxiety from constant risk alerts—one described feeling “like having a nagging parent looking over my shoulder 24/7”—suggesting that overly frequent or alarmist warnings could create stress outweighing decision support benefits. Others expressed concerns about algorithmic bias potentially discriminating against unconventional ventures that deviate from historical patterns learned by models trained on past successes, fearing that genuine innovations might receive pessimistic predictions because they lack precedent. Additionally, some students worried about data privacy despite our anonymization efforts, particularly regarding sensitive financial information and potential misuse if institutional administrators accessed individual venture data for purposes beyond research (e.g., allocating competitive funding based on predicted success probability creating self-fulfilling prophecies). These concerns highlight the need for thoughtful implementation policies governing alert frequency, prediction presentation (emphasizing uncertainty and encouraging independent judgment rather than algorithmic determinism), and strict access controls preventing misuse. Future research should longitudinally investigate psychological impacts using standardized stress and well-being measures, and develop best practices for responsible algorithmic decision support balancing analytical value against potential psychological costs.

Temporal Validity and Ecosystem Evolution: Our models trained on 2020–2024 data may degrade in predictive performance as entrepreneurship ecosystems evolve and historical patterns become obsolete, requiring ongoing maintenance and retraining that adds operational complexity. The four-year training window captures relatively stable economic conditions interrupted by COVID-19 pandemic but may not adequately represent longer-term structural shifts like technological revolutions (AI transformation, quantum computing, biotechnology breakthroughs), macroeconomic regime changes (sustained inflation, deglobalization), or regulatory reforms (data privacy laws, platform regulation). Models making predictions in 2026–2030 will increasingly extrapolate beyond their training distribution as market conditions drift from 2020 to 2024 baselines, potentially reducing accuracy over time. Addressing this temporal validity threat requires establishing monitoring systems tracking model performance degradation (comparing predictions against realized outcomes continuously) and triggering retraining when accuracy declines beyond acceptable thresholds, alongside developing online learning algorithms that continuously update model parameters as new data arrives rather than requiring complete retraining. Future research should investigate model longevity through backtesting on historical data (e.g., train on 2010–2014, test on 2015–2019 to assess prediction accuracy across temporal gaps) and develop early warning indicators predicting when models require refreshing.

## Conclusion

### Summary of principal findings

This research addressed a critical challenge in university entrepreneurship education: how to provide students with sophisticated risk assessment and decision support capabilities that compensate for their limited business experience while enhancing rather than replacing human judgment and learning. Through systematic investigation combining theoretical framework development, system implementation, and rigorous empirical validation, we established that digital twin-based intelligent decision support systems can achieve substantial improvements over conventional approaches across multiple performance dimensions. The proposed system demonstrated 94.2% prediction accuracy for identifying entrepreneurial project risks across four categories (market, financial, operational, strategic), representing 15.7% point improvement over traditional statistical methods and 8.5% point advantage over standard machine learning approaches. More importantly, the system provided early warning capabilities with average 22.1-day lead times before critical risk events materialized, sufficient temporal buffer enabling proactive interventions that traditional reactive approaches could not support. Field deployment across 15 university programs encompassing 2,847 student ventures documented that intensive system usage associated with 23.7% improvement in 24-month survival rates compared to matched control ventures not using the system—translating to approximately 101 additional surviving ventures and 323 jobs created within our experimental sample alone.

These quantitative results gain deeper significance when interpreted alongside qualitative findings from user satisfaction surveys (*N* = 412, mean satisfaction = 4.47/5.0) and semi-structured interviews (*N* = 47) revealing how students experienced and leveraged the system in practice. Student entrepreneurs particularly valued the system’s ability to identify risk patterns invisible to their limited experience (prediction accuracy rating = 4.52/5.0), provide actionable recommendations tailored to their specific circumstances rather than generic advice (recommendation relevance = 4.41/5.0), and explain the reasoning behind predictions enabling learning beyond simple compliance with algorithmic directives (trust and confidence = 4.35/5.0). However, students also surfaced important improvement opportunities, especially regarding interface design (lowest dimension score = 4.12/5.0) and mobile usability (lowest individual item = 3.87/5.0), guiding future enhancement priorities. The convergence of quantitative performance metrics and qualitative user experience data collectively support our central thesis: digital twin technology, when thoughtfully adapted to entrepreneurship contexts through specialized modeling frameworks and algorithms, can materially enhance student entrepreneurial outcomes while maintaining user acceptance and engagement essential for sustained adoption.

### Distinctive contributions to theory and practice

This investigation makes four contributions advancing both scholarly knowledge and practical capabilities at the intersection of digital twin technology, entrepreneurship education, and decision support systems, each validated through empirical evidence within our research.

Theoretical Contribution 1 - Conceptual Framework for Entrepreneurial Digital Twins (Validated: Sections II and III): We developed a novel theoretical framework extending digital twin concepts from their traditional physical-system origins (manufacturing equipment, infrastructure, vehicles) into the abstract knowledge-intensive domain of entrepreneurial ventures where the “physical entity” encompasses intangible constructs (market sentiment, team dynamics, strategic positioning) lacking direct physical measurement. This conceptual extension required reconceptualizing core digital twin principles: the “physical space” in entrepreneurial twins comprises the venture ecosystem including founders, team members, customers, competitors, and stakeholders rather than mechanical components; “synchronization” occurs through data streams capturing market signals, financial transactions, and operational metrics rather than sensor readings of temperature or vibration; “services” deliver predictive risk assessment and strategic optimization rather than predictive maintenance and production scheduling. Our state-space mathematical formalization (Eqs. 1–2 and 4–5) operationalizes these abstractions enabling rigorous computational implementation while maintaining theoretical grounding. Empirical validation demonstrated that digital twin models successfully synchronized with real venture states achieving 94.2% accuracy and early warning lead times averaging 22.1 days, confirming the viability of this conceptual framework for practical deployment.

Theoretical Contribution 2 - Specialized Machine Learning Algorithms for Student Entrepreneurship (Validated: Section III.3 and IV.2): We designed and validated three specialized machine learning algorithms—an ensemble risk classifier combining SVM, Random Forest, and LSTM networks, a multi-dimensional risk propagation model capturing cascading effects through venture networks, and a temporal risk evolution predictor forecasting future risk trajectories—specifically optimized for student entrepreneurship contexts where limited historical data, rapid pivoting behaviors, and psychological factors differentiate student ventures from established businesses. The ensemble approach achieved 94.2% accuracy outperforming individual algorithms by 3.5–4.5% points (statistically significant with *p* < 0.001, effect size d = 1.76–2.47), while ablation studies confirmed each component provided unique value justifying integration complexity. The risk propagation model contributed 5.9% points accuracy improvement over independent assessment approaches, validating our hypothesis that entrepreneurial risks exhibit systemic network characteristics requiring propagation-aware frameworks. Critically, these algorithms incorporate domain-specific features (team skill diversity, founder prior experience, university support access) and adapt to characteristics of student ventures (data scarcity, high uncertainty, frequent strategy shifts) that generic algorithms cannot effectively handle, representing methodological innovation beyond merely applying existing machine learning techniques to new domains.

Practical Contribution 3 - Integrated Intelligent Decision Support System (Validated: Section III.1 and V.3): We designed, implemented, and field-validated a complete end-to-end system architecture integrating data acquisition, digital twin modeling, risk assessment, decision optimization, and user interaction layers into a cohesive platform deployable in real university entrepreneurship programs. The system’s modular microservices architecture (eight independent services communicating via REST APIs) enables flexible deployment and scaling, while hybrid database design (PostgreSQL for structured data, MongoDB for semi-structured data, InfluxDB for time-series) optimizes storage and retrieval for heterogeneous entrepreneurship data. User satisfaction evaluation across 412 student entrepreneurs, faculty advisors, and industry mentors yielded 4.47/5.0 mean satisfaction scores with strong psychometric properties (Cronbach’s α = 0.94, seven-factor structure with excellent model fit) confirming practical usability alongside analytical performance. The system currently operates in production across 15 university programs supporting 428 + active users, demonstrating real-world viability beyond controlled experimental settings.

Empirical Contribution 4 - Rigorous Evidence for Technology-Mediated Entrepreneurship Education (Validated: Section IV.3 and V.1): We provided rare experimental evidence, combining quasi-experimental comparison with matched controls and comprehensive statistical validation, demonstrating that data-driven predictive decision support can enhance student venture outcomes—a claim frequently asserted but seldom rigorously tested in entrepreneurship education literature. The 23.7% improvement in venture success rates represents substantial practical impact (101 additional surviving ventures from 428 system users in our sample) validated through multiple statistical tests (McNemar’s χ^2^=37.45, paired t = 11.28, both *p* < 0.001, effect sizes d = 1.84–2.47 indicating very large effects). Subgroup analyses revealed system benefits distributed across venture types (technology-oriented and service-oriented ventures both showed improvements) and development stages (ideation through growth phases), supporting generalizability. Longitudinal follow-up tracking ventures through 24-month survival period ensured outcome measurement captured meaningful endpoints rather than short-term proxies, strengthening causal inference despite quasi-experimental design limitations.

These four contributions collectively advance the state of knowledge while providing immediately deployable technological capabilities addressing authentic challenges facing student entrepreneurs and university entrepreneurship programs worldwide.

### Implications for policy and practice

The demonstrated effectiveness of digital twin-based decision support systems generates several actionable policy recommendations for stakeholders involved in entrepreneurship ecosystem development.

For Higher Education Institutions: University administrators overseeing entrepreneurship programs should consider investing in digital twin decision support infrastructure as strategic priority alongside traditional mentorship and funding mechanisms, recognizing that analytical support and human guidance provide complementary rather than substitutable value. Implementation roadmap should begin with pilot deployments in select programs (1–2 year duration, 50–100 student ventures) generating performance benchmarks and user feedback informing scaled rollout decisions, rather than immediate institution-wide deployment risking costly mistakes from inadequate preparation. Successful adoption requires integrating the system into curriculum and advising workflows—not merely providing technology and assuming students will use it independently—through structured activities like mandatory risk assessment exercises, advisor discussions of system recommendations, and reflection assignments examining prediction accuracy to foster critical engagement rather than passive compliance with algorithmic outputs.

For Government Entrepreneurship Policy: Policy makers designing public entrepreneurship support programs should expand intervention portfolios beyond capital provision and regulatory simplification to include analytical decision support, recognizing that venture success depends on both resource availability and resource allocation quality. Potential implementation mechanisms include: (1) conditioning entrepreneurship grants on participation in structured decision support programs ensuring public investments receive analytical guidance maximizing success probability, (2) subsidizing university development or licensing of decision support systems reducing institutional cost barriers, (3) establishing national entrepreneurship data infrastructure enabling researchers and technology developers to access aggregated anonymized venture data for training improved prediction models while protecting individual venture confidentiality. International development agencies supporting entrepreneurship in emerging economies should particularly prioritize decision support infrastructure recognizing that entrepreneurs in resource-constrained contexts face heightened risk from decision errors yet lack access to sophisticated advisors available in developed ecosystems.

For Entrepreneurship Educators and Researchers: Scholars investigating entrepreneurship education effectiveness should incorporate digital twin and machine learning methodologies into their research toolkits, recognizing these technologies enable research designs previously infeasible with traditional methods. For instance, digital twin longitudinal tracking generates detailed behavioral data (venture decisions, strategy pivots, resource allocation changes) at temporal granularity (daily or hourly updates) and sample sizes (thousands of ventures) enabling investigation of micro-level decision processes and rare event dynamics that survey-based research cannot capture. However, researchers must balance analytical opportunities against ethical responsibilities: extensive data collection powering digital twin fidelity creates surveillance capabilities requiring robust informed consent, institutional review board oversight, and data governance frameworks protecting student privacy. Professional organizations should develop ethical guidelines for responsible entrepreneurship analytics addressing algorithmic bias, prediction transparency, and appropriate uses of decision support technologies.

### Future research directions and development prospects

While this research establishes foundational viability of digital twin-based entrepreneurship decision support, numerous opportunities exist for extending and enhancing the approach through technological advancement and theoretical refinement.

Advanced AI Integration: Future system generations should incorporate cutting-edge artificial intelligence capabilities emerging from recent research breakthroughs but not available during our development phase. Large language models (LLMs) such as GPT-4, Claude, and domain-specialized variants trained on entrepreneurship corpora could enable natural language interaction where students describe challenges conversationally (“our customer acquisition is slower than expected and we’re worried about runway”) and receive contextual advice in narrative form rather than numerical risk scores, making the system more accessible to non-technical users. Reinforcement learning algorithms could optimize sequential decision-making under uncertainty, learning policies for when to pivot versus persevere, when to seek funding versus bootstrap, when to expand team versus maintain lean operations through trial-and-error interaction with venture environment. Causal inference methods including do-calculus and counterfactual reasoning could move beyond correlational prediction toward prescriptive guidance answering “what would happen if I made decision X versus decision Y?” with causal rather than merely associative confidence. Explainable AI (XAI) techniques such as SHAP values and attention visualization could make black-box model predictions interpretable for educational contexts where students need to understand algorithmic reasoning to learn from recommendations rather than simply comply with them.

Domain-Specific Model Development: Our current system provides generic risk assessment applicable across diverse venture types, but future work should develop industry-specialized models capturing sector-specific risk patterns and decision logics. For example, biotechnology ventures face distinctive challenges (regulatory approval timelines, IP protection complexities, capital-intensive R&D) fundamentally different from software ventures (rapid iteration, network effects, “winner-take-all” dynamics), suggesting that specialized models incorporating domain knowledge could outperform generic approaches. Academic research should investigate modular model architectures with shared base components capturing universal entrepreneurship principles (team dynamics, financial management) and swappable specialized modules encoding sector-specific factors (FDA approval processes for biotech, app store rankings for mobile software), enabling efficient development of industry-tailored variants without complete redesign. Additionally, models should extend beyond traditional for-profit entrepreneurship to social enterprises, impact ventures, academic spin-offs, and intrapreneurship within established organizations—each representing distinctive contexts with unique risk profiles and success metrics requiring specialized treatment.

Cross-Cultural and International Adaptation: Given our dataset’s concentration in Chinese university contexts, systematic international validation and cultural adaptation represents crucial next steps for global deployment. Researchers should conduct cross-national studies examining: (1) how entrepreneurship risk factors and their relative importance vary across cultural contexts (e.g., whether team diversity matters more in individualistic Western cultures versus collectivist Asian cultures), (2) whether prediction models trained on one nation’s data generalize to other nations or require local retraining, (3) how regulatory differences across countries (intellectual property protection, labor laws, tax policies) affect risk patterns necessitating country-specific model components. Transfer learning approaches enabling models to adapt from data-rich source countries to data-scarce target countries with minimal local training data could accelerate international deployment while maintaining prediction quality. Anthropological and sociological perspectives should inform cultural adaptation beyond technical model adjustment—for instance, investigating how cultural attitudes toward failure, risk, and technology influence system acceptance and usage patterns, and whether interface design and interaction paradigms should vary across cultures.

Blockchain Integration for Trust and Data Sovereignty: Emerging blockchain technologies offer potential solutions to data privacy and trust challenges that could enhance user confidence in digital twin systems. Implementing venture data storage on permissioned blockchain networks with cryptographic access controls could provide students with verifiable data sovereignty—mathematical proof that their sensitive business information remains encrypted and inaccessible to unauthorized parties including system operators, while still enabling aggregated anonymized analytics for model training through secure multi-party computation protocols. Smart contracts could automate data access agreements ensuring that system operators only access student data for explicitly consented purposes with automatically enforced usage restrictions, addressing the surveillance concerns some students expressed. Additionally, blockchain-verified audit trails documenting all prediction and recommendation history could provide accountability mechanisms enabling students to retrospectively review system guidance and developers to investigate prediction errors. Academic research should empirically investigate whether blockchain integration actually enhances user trust and adoption beyond theoretical privacy guarantees, as the technical complexity might create usability barriers offsetting transparency benefits.

Longitudinal Impact Studies: While our research documented improvements in 24-month venture survival rates, longer-term impacts on student entrepreneurs’ careers and well-being remain unknown and merit investigation through extended longitudinal tracking. Future studies should follow student entrepreneurs 5–10 years post-graduation examining: (1) whether early system exposure improves subsequent entrepreneurial endeavors (serial entrepreneurs), (2) how system experience influences career trajectories for students whose initial ventures failed (do they re-enter entrepreneurship or avoid it?), (3) whether analytical decision-making skills cultivated through system interaction transfer to non-entrepreneurial career contexts (corporate positions, policy roles), and (4) psychological long-term impacts such as entrepreneurial self-efficacy, risk tolerance, and stress resilience. Such longitudinal research would clarify whether benefits represent temporary performance boosts or deeper capability development persisting beyond immediate system usage, informing cost-benefit analyses for institutional adoption decisions.

### Concluding remarks

University student entrepreneurship represents a frontier where educational missions intersect with economic development imperatives and individual aspirations for self-directed careers. The high failure rates plaguing student ventures—consequences of limited experience, resource constraints, and rapidly changing markets—create both human costs (dashed dreams, financial losses, psychological trauma) and societal opportunity costs (innovations unrealized, jobs uncreated, economic dynamism diminished). This research demonstrates that thoughtfully designed technological interventions leveraging digital twin frameworks and machine learning can materially improve student entrepreneurial outcomes, not by replacing human judgment but by augmenting it through analytical capabilities complementing students’ creativity and passion with pattern recognition derived from thousands of prior ventures. The 94.2% prediction accuracy, 22.1-day early warning lead times, and 23.7% success rate improvements documented in our rigorous empirical evaluation provide compelling evidence that the vision of “augmented entrepreneurship”—human-AI collaboration where technology handles quantitative pattern analysis while humans focus on relationships, creativity, and ethical judgment—represents not speculative futurism but achievable reality with current technologies.

Yet we must approach this technological optimism with balanced realism, acknowledging both possibilities and pitfalls. Digital twin systems are powerful tools but not panaceas—they cannot create innovative ideas, substitute for passionate execution, or overcome fundamental market impossibilities where customer demand simply does not exist regardless of execution excellence. Moreover, as our discussion of limitations clarified, the systems introduce new challenges around data privacy, algorithmic bias, psychological pressure from continuous monitoring, and equity concerns about digital divides between well-resourced institutions able to deploy sophisticated systems and under-resourced programs unable to afford them. Realizing the technology’s potential while managing its risks requires ongoing collaboration among computer scientists, entrepreneurship scholars, educators, policy makers, and most importantly, student entrepreneurs themselves whose feedback should guide system evolution.

The research journey underlying this dissertation has convinced me that we stand at an inflection point in entrepreneurship education where data-driven decision support technologies transition from laboratory curiosities to practical necessities. Just as GPS navigation systems revolutionized transportation by democratizing route optimization previously requiring expert local knowledge, entrepreneurial decision support systems promise to democratize business judgment previously monopolized by experienced entrepreneurs and professional investors. The question is no longer whether such systems can work—our results demonstrate they can—but rather how to deploy them responsibly, equitably, and effectively in service of the millions of students worldwide who dream of building businesses that matter. This research provides both empirical foundation and practical blueprint for answering that question, but the work has only begun. Future researchers and practitioners will extend, refine, and surpass this initial contribution, collectively building an ecosystem where every student entrepreneur, regardless of prior experience or institutional resources, can access world-class analytical guidance supporting their entrepreneurial journeys. That future, though not yet fully realized, feels tantalizingly within reach—and getting there represents a worthy goal to which this research aspires to contribute^100–111^.

## Data Availability

Due to the sensitive nature of student entrepreneurial venture data containing personally identifiable information and proprietary business details, the raw dataset cannot be publicly shared without compromising participant privacy protections. To support research reproducibility, we will provide complete source code for all data preprocessing pipelines, machine learning algorithms, digital twin modeling components, statistical analysis scripts, comprehensive documentation, tutorial notebooks, pretrained model weights, and synthetic datasets matching the distributional properties of our actual data through a GitHub repository upon paper publication. Researchers requiring access to the full dataset for legitimate academic purposes may contact the corresponding author to initiate institutional data sharing procedures subject to ethics committee approval.
